# The differential diagnosis value of radiomics-based machine learning in Parkinson’s disease: a systematic review and meta-analysis

**DOI:** 10.3389/fnagi.2023.1199826

**Published:** 2023-07-06

**Authors:** Jiaxiang Bian, Xiaoyang Wang, Wei Hao, Guangjian Zhang, Yuting Wang

**Affiliations:** ^1^School of Clinical Medicine, Weifang Medical University, Weifang, China; ^2^Department of Neurosurgery, Weifang People’s Hospital, Weifang, China

**Keywords:** Parkinson’s disease, radiomics, machine learning, diagnostic accuracy, meta-analysis, systematic review

## Abstract

**Background:**

In recent years, radiomics has been increasingly utilized for the differential diagnosis of Parkinson’s disease (PD). However, the application of radiomics in PD diagnosis still lacks sufficient evidence-based support. To address this gap, we carried out a systematic review and meta-analysis to evaluate the diagnostic value of radiomics-based machine learning (ML) for PD.

**Methods:**

We systematically searched Embase, Cochrane, PubMed, and Web of Science databases as of November 14, 2022. The radiomics quality assessment scale (RQS) was used to evaluate the quality of the included studies. The outcome measures were the c-index, which reflects the overall accuracy of the model, as well as sensitivity and specificity. During this meta-analysis, we discussed the differential diagnostic value of radiomics-based ML for Parkinson’s disease and various atypical parkinsonism syndromes (APS).

**Results:**

Twenty-eight articles with a total of 6,057 participants were included. The mean RQS score for all included articles was 10.64, with a relative score of 29.56%. The pooled c-index, sensitivity, and specificity of radiomics for predicting PD were 0.862 (95% CI: 0.833–0.891), 0.91 (95% CI: 0.86–0.94), and 0.93 (95% CI: 0.87–0.96) in the training set, and 0.871 (95% CI: 0.853–0.890), 0.86 (95% CI: 0.81–0.89), and 0.87 (95% CI: 0.83–0.91) in the validation set, respectively. Additionally, the pooled c-index, sensitivity, and specificity of radiomics for differentiating PD from APS were 0.866 (95% CI: 0.843–0.889), 0.86 (95% CI: 0.84–0.88), and 0.80 (95% CI: 0.75–0.84) in the training set, and 0.879 (95% CI: 0.854–0.903), 0.87 (95% CI: 0.85–0.89), and 0.82 (95% CI: 0.77–0.86) in the validation set, respectively.

**Conclusion:**

Radiomics-based ML can serve as a potential tool for PD diagnosis. Moreover, it has an excellent performance in distinguishing Parkinson’s disease from APS. The support vector machine (SVM) model exhibits excellent robustness when the number of samples is relatively abundant. However, due to the diverse implementation process of radiomics, it is expected that more large-scale, multi-class image data can be included to develop radiomics intelligent tools with broader applicability, promoting the application and development of radiomics in the diagnosis and prediction of Parkinson’s disease and related fields.

**Systematic review registration:**

https://www.crd.york.ac.uk/PROSPERO/display_record.php?RecordID=383197, identifier ID: CRD42022383197.

## 1. Introduction

Parkinson’s disease (PD) is the second utmost common neurodegenerative illness, and its prevalence is anticipated to more than double over the next 30 years (GBD 2016 Parkinson’s Disease Collaborators, 2018; Tolosa et al., 2021). The increasing number of patients will impose a significant medical and economic burden on society. Currently, the diagnosis of PD depends on a set of standards proposed by the International Parkinson and Movement Disorder Society (MDS) in 2015 (Postuma et al., 2015). During this process, clinicians rely on limited support and exclusion criteria, as well as “Red flags” to evaluate patients, which is time-consuming and labor-intensive and is related to the experience of clinical experts. Moreover, in the early stages, it is challenging to accurately and timely identify PD due to overlapping symptoms with atypical Parkinson’s syndrome (APS) (Respondek et al., 2019). Studies have shown that about 20–30% of patients with multiple system atrophy (MSA) or progressive supranuclear palsy (PSP) were initially misdiagnosed as idiopathic Parkinson’s disease (IPD) in clinical practice (Saeed et al., 2020). In addition, in terms of the motor subtypes of PD, the postural instability and gait difficulty subtype (PIGD) has greater damage to the neurological function than the tremor-dominant subtype (TD) and has a relatively poor response to deep brain stimulation (DBS) and levodopa therapy (Sun et al., 2021). Given the above reasons, early and accurate identification of PD and differentiation of its subtypes have profound clinical significance for developing individualized treatment plans and predicting prognosis.

Radiomics has emerged as a result of the development of artificial intelligence and medical precision. It extracts high-dimensional data from clinical images (such as PET, MRI, and CT) that can be mined (Lambin et al., 2012, 2017). Through analyzing and constructing classification models, radiomics can be utilized alone or in conjunction with histological, demographic, genomic, or proteomic data to support evidence-based clinical decision-making (Rizzo et al., 2018). In recent years, radiomics has gradually demonstrated significant clinical utility in the diagnosis, differential diagnosis, severity assessment, and prediction of disease progression in Parkinson’s disease (PD), Parkinson’s syndrome, and other neurodegenerative disorders, through the utilization of various imaging techniques (Adeli et al., 2016; Klyuzhin et al., 2016; Rahmim et al., 2016).

However, radiomics encompasses diverse methods in its implementation and is highly correlated with the expertise of clinical experts. The diagnostic performance of radiomics needs to be comprehensively evaluated from an evidence-based perspective. Systematic reviews, as a component of evidence-based medicine, can provide relevant guidance to some extent in formulating clinical strategies. Therefore, we conducted this study to evaluate the accuracy of radiomics-based machine learning in diagnosing Parkinson’s disease (PD) and to summarize some of the challenges currently faced by radiomics in order to provide a reference for future applications of radiomics.

## 2. Materials and methods

Our systematic review and meta-analysis were conducted based on the Preferred Reporting Items for Systematic Reviews and Meta-Analyses (PRISMA 2020) guidelines (Moher et al., 2009). The PRISMA guidelines are provided in Supplementary Table 1. This study was registered on PROSPERO (ID: CRD42022383197).

### 2.1. Inclusion and exclusion criteria

#### 2.1.1. Inclusion criteria

(1) Patients clinically diagnosed with Parkinson’s disease (PD) with complete imaging data.
(2) Fully constructed radiomics ML models for the diagnosis of PD.
(3) Studies without external validation were also included.
(4) Published studies employing the same or different machine learning (ML) algorithms on a single dataset.
(5) Studies reported in English were included.

#### 2.1.2. Exclusion criteria

(1) Meta-analyses, reviews, guidelines, expert opinions, etc.
(2) Studies that only performed differential factor analysis and did not construct a complete ML model.
(3) Studies that lacked outcome indicators for ML model prediction accuracy (Roc, c-statistic, c-index, sensitivity, specificity, accuracy, recall, precision, confusion matrix, diagnostic four-grid table, F1 score, calibration curve).

### 2.2. Literature search strategy

We performed a comprehensive search of the PubMed, Cochrane, Embase, and Web of Science databases for all available literature up to November 14th, 2022, utilizing a combination of subject headings and free-text terms. Our search was not restricted by language or geographic region. The detailed search strategy is shown in Supplementary Table 2.

### 2.3. Study selection and data extraction

We imported the retrieved literature into EndNote and removed duplicate articles. The remaining articles were screened based on their titles and abstracts. For the potentially relevant studies, we downloaded and read the full-text articles to determine their eligibility according to the inclusion and exclusion criteria. Before extracting the data, a standardized electronic spreadsheet was developed. The extracted information included the title, first author, publication year, country, study type, patient source, PD diagnostic criteria, radiomics source, whether complete imaging protocols were recorded, number of imaging reviewers involved, whether pre-experiments were conducted under different imaging parameters, whether repeated measurements were performed at different times, imaging segmentation software, texture extraction software, number of PD cases/images, total number of cases/images, number of PD cases/images in the training set, number of cases/images in the training set, method of generating the validation set, number of PD cases in the validation set, number of cases in the validation set, variable selection method, type of model used, modeling variables, whether radiomics scores were constructed, overfitting evaluation, whether the code and data were made publicly available, and model evaluation indications.

The literature screening and data extraction were independently conducted by two researchers (JB and XW), and cross-checking was performed afterward. In cases of disagreement, a third researcher (WH) was consulted to resolve the issue.

### 2.4. Quality assessment

The methodological quality of the included studies was assessed by the two researchers (JB and XW) using the Radiomics Quality Score (RQS), and an interactive check was conducted afterward (Lambin et al., 2012). If there was a dispute, a third researcher (WH) was asked to assist in the decision-making process. RQS is a radiomics-specific quality assessment tool that scores the quality of the original study design based on 16 items (e.g., whether the image acquisition method and data were described in detail, whether measures were taken to prevent overfitting or multiple segmentation, whether the study was prospective, and whether the model was validated and how it was validated). Each criterion is assigned a numerical value that corresponds to the impact of the study on radiomics research, and the total score ranges from −8 to 36, which is then converted into a percentage score (0–100%). This score represents the rigor of model development and the evaluation of the study’s impact on the field.

### 2.5. Outcome measures

The primary outcome measure of our systematic review is the c-index, which reflects the overall accuracy of the ML model. However, when there is a severe imbalance in the number of cases between the observation group and the control group, the c-index may not be sufficient to reflect the accuracy of the ML model for disease diagnosis. As a result, our primary outcome measures also included sensitivity and specificity.

### 2.6. Statistical analysis

Our analysis consists of three parts: (a) Diagnosis of Parkinson’s disease [comparing PD patients and healthy controls (HC)], (b) Differential diagnosis of Parkinson’s disease (comparing idiopathic PD patients and APS patients), and (c) Parkinson’s disease subtypes (comparing TD and PIGD). This study reported the c-index with a 95% confidence interval (CI), which reflected the accuracy of ML models. In cases where the original literature lacks a 95% confidence interval or standard error of the c-index, they were estimated by the formula proposed by Debray et al. (2019). The meta-analysis of sensitivity and specificity requires the diagnostic fourfold table (true negatives, true positives, false negatives, and false positives), but few original studies directly reported a diagnostic fourfold table. Thus, we need to calculate the fourfold table by combining sensitivity and specificity with the number of cases. However, in cases where sensitivity and specificity are missing, Origin 2020 was used to extract them from the ROC curve.

A random effects model was used to perform the meta-analysis of the overall accuracy of the ML model, as reflected by the c-index, while a bivariate mixed effects model was used for the meta-analysis of the sensitivity and specificity (Reitsma et al., 2005). Statistical analysis was performed using Stata 15.0 (Stata Corporation, USA). A *p*-value < 0.05 was considered statistically significant.

## 3. Results

### 3.1. Study selection

**FIGURE 1:**
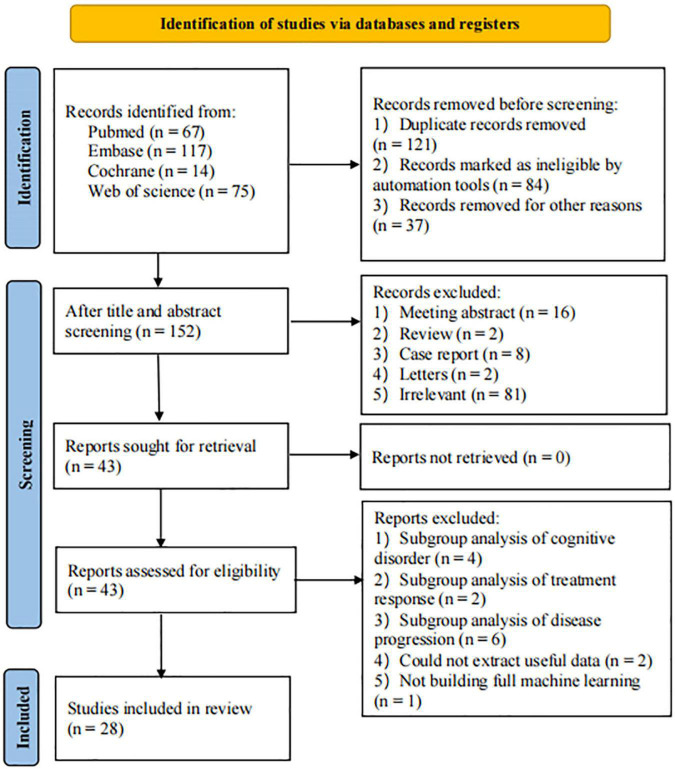
Literature screening flowchart.

Figure 1 illustrates the PRISMA flow diagram of the study selection. The search identified 67 studies from PubMed, 117 studies from Embase, 14 studies from Cochrane, and 75 studies from Web of Science. Following the exclusion of 121 duplicate studies, 43 studies were screened based on their titles or abstracts. Ultimately, a total of 28 articles (Cheng et al., 2019; Shinde et al., 2019; Wu et al., 2019; Xiao et al., 2019; Cao et al., 2020, 2021; Liu et al., 2020; Pang et al., 2020, 2022; Shu et al., 2020; Dhinagar et al., 2021; Hu et al., 2021; Li et al., 2021, 2022; Ren et al., 2021; Shi et al., 2021, 2022a, 2022b; Sun et al., 2021, 2022; Tupe-Waghmare et al., 2021; Zhang et al., 2021; Ben Bashat et al., 2022; Guan et al., 2022; Kang et al., 2022; Kim et al., 2022; Shiiba et al., 2022; Zhao et al., 2022) were deemed eligible and included in this meta-analysis.

### 3.2. Study characteristics

The characteristics of the studies included in this research are shown in Table 1 and Supplementary Table 4. The original 28 studies were published between 2019 and 2022, with 27 of them from Asia (Cheng et al., 2019; Shinde et al., 2019; Wu et al., 2019; Xiao et al., 2019; Cao et al., 2020, 2021; Liu et al., 2020; Pang et al., 2020, 2022; Shu et al., 2020; Hu et al., 2021; Li et al., 2021, 2022; Ren et al., 2021; Shi et al., 2021, 2022a, 2022b; Sun et al., 2021, 2022; Tupe-Waghmare et al., 2021; Zhang et al., 2021; Ben Bashat et al., 2022; Guan et al., 2022; Kang et al., 2022; Kim et al., 2022; Shiiba et al., 2022; Zhao et al., 2022) and one from North America (Dhinagar et al., 2021). The study comprised a total of 6,057 participants, with 3,422 patients diagnosed with PD, 1,983 healthy controls, and 652 cases of APS (476 with MSA and 176 with PSP). Among these studies, 22 focused on the diagnosis of PD (Cheng et al., 2019; Shinde et al., 2019; Wu et al., 2019; Xiao et al., 2019; Cao et al., 2020, 2021; Liu et al., 2020; Shu et al., 2020; Dhinagar et al., 2021; Li et al., 2021, 2022; Ren et al., 2021; Shi et al., 2021, 2022a, 2022b; Sun et al., 2021, 2022; Zhang et al., 2021; Ben Bashat et al., 2022; Guan et al., 2022; Kang et al., 2022; Shiiba et al., 2022), while six studies focused on the differential diagnosis of PD and APS (Pang et al., 2020, 2022; Hu et al., 2021; Tupe-Waghmare et al., 2021; Kim et al., 2022; Zhao et al., 2022). In addition, two studies addressed the differential diagnosis of PD with or without depression (Li et al., 2021; Zhang et al., 2021), and one study fixated on the differential diagnosis of TD and PIGD (Sun et al., 2021). There were 14 ML models, including SVM (Support Vector Machine), CNN (Convolutional Neural Network), LR (Logistic Regression), LDA (Linear Discriminant Analysis), RF (Random Forest), LASSO (Least Absolute Shrinkage and Selection Operator), DT (Decision Tree), KNN (K-Nearest Neighbor), ANN (Artificial Neural Network), GNB (Gaussian Naive Bayes), GP (Gaussian Process), Bayes (Bayesian Network), ADA (Adaptive Boosting), and QDA (Quadratic Discriminant Analysis).

**TABLE 1:** (A) Basic characteristic of the included studies; (B) Modeling information for included studies.

Schedule A							
**No.**	**References**	**Country**	**Research type**	**Differential diagnosis**	**Patient source**	**Diagnostic criteria for Parkinson’s disease**	**Radiomics source**
1	Zhao et al., 2022	China	Case-control	PD vs. APS	Single center	The MDS PD Criteria	PET
2	Sun et al., 2022	China	Case-control	PD vs. HCs	Multi-center	The MDS PD Criteria	PET
3	Shiiba et al., 2022	Japan	Case-control	PD vs. HCs	Registration database	Not described	SPECT
4	Shi et al., 2022b	China	Case-control	PD vs. HCs	Registration database	Not described	MRI
5	Shi et al., 2022a	China	Case-control	PD vs. HCs	Registration database	Not described	MRI
6	Pang et al., 2022	China	Case-control	PD vs. MSA-p	Single center	Not described	MRI
7	Li et al., 2022	China	Case-control	PD vs. HCs	Single center	The MDS PD Criteria	MRI
8	Kim et al., 2022	Republic of Korea	Case-control	PD vs. MSA-p PD vs. MSA-c PD vs. PSP	Single center	The UK PD SBB criteria	MRI
9	Kang et al., 2022	China	Case-control	PD vs. HCs	Single center	The MDS PD Criteria	MRI
10	Guan et al., 2022	China	Case-control	PD vs. HCs	Single center	The UK PD SBB criteria	MRI
11	Ben Bashat et al., 2022	Israel	Case-control	PD vs. HCs	Single center	The MDS PD Criteria	MRI/SPECT
12	Zhang et al., 2021	China	Case-control	DPD vs. HCs NDPD vs. HCs DPD vs. NDPD	Single center	Not described	MRI
13	Tupe-Waghmare et al., 2021	India	Case-control	PD vs. HCs PD vs. APS	Single center	The UK PD SBB criteria	MRI
14	Sun et al., 2021	China	Case-control	PIGD vs. HCs TD vs. HCs PIGD vs. TD	Registration database	Not described	MRI
15	Shi et al., 2021	China	Case-control	PD vs. HCs	Registration database	The UK PD SBB criteria	MRI
16	Ren et al., 2021	China	Case-control	PD vs. HCs	Single center	The MDS PD Criteria	MRI
17	Li et al., 2021	China	Case-control	PD vs. HCs PD vs. DPD	Single center	The MDS PD Criteria	MRI
18	Hu et al., 2021	China	Case-control	PD vs. MSA	Single center	The MDS PD Criteria	MRI/PET
19	Dhinagar et al., 2021	United States	Case-control	PD vs. HCs	Registration database	Not described	MRI
20	Cao et al., 2021	China	Case-control	PD vs. HCs	Single center	Not described	MRI
21	Shu et al., 2020	China	Case-control	PD vs. HCs	Registration database	Not described	MRI
22	Pang et al., 2020	China	Case-control	PD vs. MSA-p	Single center	The UK PD SBB criteria	MRI
23	Liu et al., 2020	China	Case-control	PD vs. HCs	Single center	The UK PD SBB criteria	MRI
24	Cao et al., 2020	China	Case-control	PD vs. HCs	Single center	Not described	MRI
25	Xiao et al., 2019	China	Case-control	PD vs. HCs	Single center	Not described	MRI
26	Wu et al., 2019	China	Cohort study	PD vs. HCs	Multi-center	The UK PD SBB criteria	PET
27	Shinde et al., 2019	India	Case-control	PD vs. HCs PD vs. APS	Single center	The UK PD SBB criteria	MRI
28	Cheng et al., 2019	China	Case-control	PD vs. HCs	Single center	The UK PD SBB criteria	MRI
**Schedule B**							
**No.**	**References**	**Total sample size**	**Sample size in training set**	**Verification method**	**Sample size in validation set**	**Variable screening method**	**Type of model**
1	Zhao et al., 2022	1017 (IPD 682, MSA 168, PSP 124, HCs 43)	737	External validation	280	Not described	CNN
2	Sun et al., 2022	406 (PD 125, HCs 281)	358	External validation	48	Not described	SVM, CNN
3	Shiiba et al., 2022	413 (PD 312, HCs 101)	224	Random sampling External validation	189	LASSO	SVM, KNN, LDA, DT
4	Shi et al., 2022b	143 (PD 86, HCs 57)	100	External validation	43	*T*-test, LASSO	SVM
5	Shi et al., 2022a	213 (PD123, HCs90)	213	fivefold cross validation 10-fold cross validation	–	*T*-tests, RFE	SVM
6	Pang et al., 2022	152 (PD 77, MSA-p 75)	107	Random sampling	45	LASSO, mRMR	SVM
7	Li et al., 2022	110 (PD 56, HCs 54)	60	External validation	50	LASSO	LR
8	Kim et al., 2022	128 (PD 56, MSA-p 34, MSA-c 21, PSP 17)	90 (PD 39 vs. MSA-P 24) (PD 39 vs. MSA-c 15) (PD 39 vs. PSP 12)	Random sampling	38 (PD 17 vs. MSA-P 10) (PD 17 vs. MSA-c 6) (PD 17 vs. PSP 5)	Autocorrelation and fisher score algorithm	KNN, SVM, GP, RF, DT, MLP, ADA, GNB, QDA
9	Kang et al., 2022	149 (PD 104, HCs 45)	104	Random sampling	45	LASSO	MLR, SVM
10	Guan et al., 2022	350 (PD 171, HCs 179)	244	External validation	106	RF	RF
11	Ben Bashat et al., 2022	127 (PD 46, HCs 81)	127	fivefold cross validation	–	PCA	SVM
12	Zhang et al., 2021	120 (PD 70, HCs 50)	84	Random sampling	36	LASSO	LASSO, RF, SVM
13	Tupe-Waghmare et al., 2021	201 (PD 65, APS 61 (MSA 31, PSP 30), HCs 75)	160	Random sampling	41 (PD 13 vs. HCs 15) (PD 13 vs. APS 13)	RFECV	RF
14	Sun et al., 2021	230 (PD 134, HCs 96)	185	Random sampling	45	LASSO	SVM, LR, MLP
15	Shi et al., 2021	100 (PD 59, HCs 41)	80	Random sampling	20	*T*-test, LASSO	LASSO
16	Ren et al., 2021	190 (PD 95, HCs 95)	126	Random sampling	64	LASSO, mRMR	RF, SVM, KNN, LR
17	Li et al., 2021	164 (PD 82, HCs 82)	164	fivefold cross validation	–	LASSO, Pearson correlation analyses, Multivariate analyses	LR
18	Hu et al., 2021	90 (PD 60, MSA 30)	63	Random sampling	27	LASSO, mRMR	LASSO, LR
19	Dhinagar et al., 2021	588 (PD 445, HCs 143)	424	Random sampling External validation	164	RFE	RF, CNN
20	Cao et al., 2021	116 (PD 67, HCs 49)	71	Random sampling	45	Mann-Whitney *U*-test	LR
21	Shu et al., 2020	336 (PD 168, HCs 168)	234	Random sampling	102	LASSO, mRMR, GBDT	SVM, Bayes, LR, RF, DT
22	Pang et al., 2020	185 (PD 83, MSA-p 102)	129	Random sampling	56	*T*-tests, LASSO	SVM
23	Liu et al., 2020	138 (PD 69, HCs 69)	96	Random sampling	42	LASSO	LASSO
24	Cao et al., 2020	116 (PD 67, HCs 49)	93	Random sampling	23	Mann-Whitney *U*-test, LASSO	SVM, RF
25	Xiao et al., 2019	140 (PD 87, HCs 53)	140	sevenfold cross validation	–	ICC, RFE	LR, SVM, CNN
26	Wu et al., 2019	230 (PD 113, HCs 117)	146	Random sampling External validation	84	Autocorrelation and fisher score algorithm	SVM, RF
27	Shinde et al., 2019	100 (PD 45, APS 20 (MSA 15, PSP 5), HCs 35)	69 (PD 30 vs. HCs 25) (PD 30 vs. APS 14)	Random sampling	31 (PD 15 vs. HCs 10) (PD 15 vs. APS 6)	Average information gain	RF, CNN
28	Cheng et al., 2019	164 (PD 87, HCs 77)	164	threefold cross validation	–	ANOVA, RF, RFE	SVM

**(A)** The MDS PD criteria: the movement disorder society PD criteria; the UK PD SBB criteria: the UK PD society brain bank criteria.

**(B)** In article 8, 13, and 27, different research objects were used for training and verification, and the specific sample numbers were listed in the Table 2. In article 5, 11, 17, 25, and 28, the method of cross-validation is adopted, so there is no specific sample number of validation set 3. Article 5 used the same dataset of articles 4 and 15. Articles 20 and 24 were based on the same dataset. PCA, principle component analysis; RFE, recursive feature elimination; RFECV, recursive feature elimination with cross-validation; ICC, intraclass correlation coefficient; ANOVA, analysis of variance; MSA-c, multiple system atrophy-cerebellar type; MSA-p, multiple system atrophy-parkinsonian type.

### 3.3. Quality analysis

**FIGURE 2:**
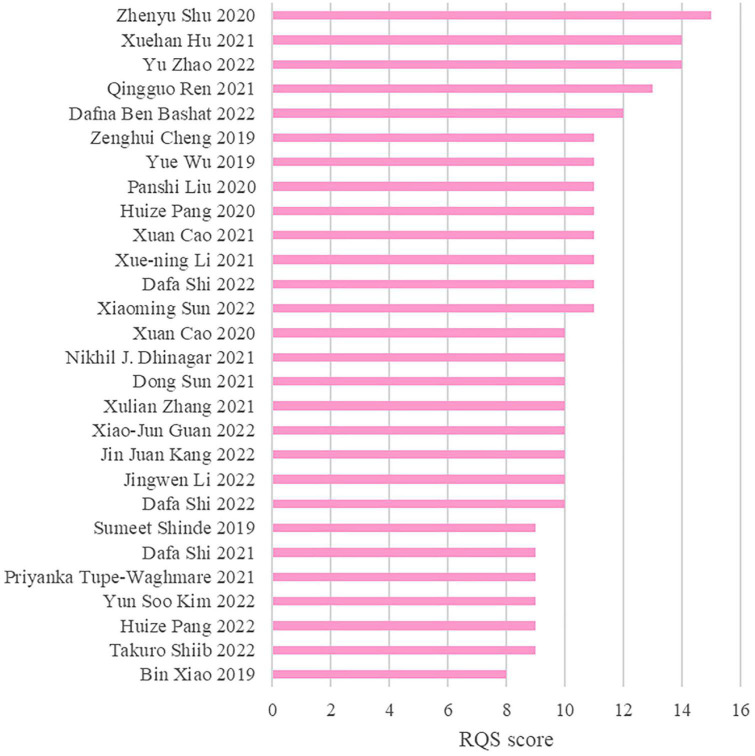
Bar chart (quality evaluation table).

Figure 2 illustrates the RQS scores and relative scores of all 28 studies included in this research, as evaluated by the two reviewers (JB and XW). The mean RQS score for the studies was 10.64 (range 8–15), while the mean relative score was 29.56% (range 22.22–41.67%). All the studies reported well-documented image acquisition protocols and performed feature selection and data dimensionality reduction to reduce model overfitting. For model evaluation, most studies provided discriminant statistics (e.g., ROC curve, c-index, AUC) and their statistical significance (e.g., *p*-value, confidence interval), while calibration statistics were less frequently mentioned. Ten studies (Cao et al., 2020, 2021; Pang et al., 2020, 2022; Shu et al., 2020; Hu et al., 2021; Li et al., 2021; Zhang et al., 2021; Sun et al., 2022; Zhao et al., 2022) conducted multivariate analyzes of non-radiomics features, such as plasma FAM19A5, demographic and clinical characteristics, impaired sense of smell, and cognitive impairment, which provided more comprehensive integrated models. One study (Ben Bashat et al., 2022) also examined and discussed biological correlations; demonstrating phenotypic differences that could be related to underlying gene-protein expression patterns broadens the perception of radiomics and biology. Nine studies (Liu et al., 2020; Shu et al., 2020; Cao et al., 2021; Ren et al., 2021; Shi et al., 2021; Guan et al., 2022; Li et al., 2022; Shiiba et al., 2022; Zhao et al., 2022) conducted cut-off value analysis to assess the risk of model diagnostic prediction accuracy. However, only five studies evaluated the potential clinical utility of the model by decision curve analysis (Wu et al., 2019; Shu et al., 2020; Hu et al., 2021; Ren et al., 2021; Zhao et al., 2022), and none performed a cost-effectiveness analysis. Since there is currently no clear gold standard for the clinical diagnosis of PD, it is challenging to evaluate the degree of consistency between the model and the current “gold standard” method.

Only one study has compared the diagnostic accuracy of ML models based on magnetic resonance imaging (MRI) with those based on dopamine transporter single-photon emission tomography (DAT-SPECT) imaging (Ben Bashat et al., 2022). Additionally, only two studies have prospectively validated the use of radiomic biomarkers (Sun et al., 2022; Zhao et al., 2022). No studies have investigated the stability of radiomics signatures across different scanners or time points. In terms of open science and data, most studies do not provide open-source code directly. The quality evaluation scores are shown in Supplementary Table 3.

### 3.4. Meta-analysis

#### 3.4.1. Diagnosis of PD

In terms of the diagnosis of PD, 42 ML models in the training set reported a c-index, with a pooled c-index of 0.862 (95% CI: 0.833–0.891). In the validation set, 78 ML models reported a c-index, with a pooled c-index of 0.871 (95% CI: 0.853–0.890).

**FIGURE 3:**
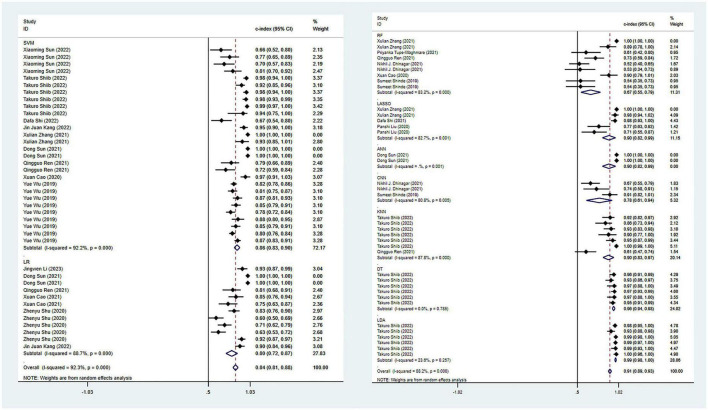
Meta-analysis results of c-index for PD diagnosis based on radiomics-based machine learning (Validation set). Due to the large amount of relevant data involved, the results of the verification set are presented in two parts, and the forest plot for the training set is provided in the Supplementary material.

There were 42 fourfold tables for diagnosis that were available and could be directly or indirectly extracted in the training set, and the pooled sensitivity and specificity were 0.91 (95% CI: 0.86–0.94) and 0.93 (95% CI: 0.87–0.96), respectively. There were 60 models in the validation set, and the sensitivity and specificity for disease diagnosis were 0.86 (95% CI: 0.81–0.89) and 0.87 (95% CI: 0.83–0.91), respectively, as depicted in Figure 3, Table 2 and Supplementary Table 5.

**TABLE 2:** Meta-analysis results of sensitivity and specificity for PD diagnosis based on radiomics-based machine learning.

Model	Training set					Validation set				
	**Number**	**Sen (95% CI)**	***I*^2^ (%)**	**Spe (95% CI)**	***I*^2^ (%)**	**Number**	**Sen (95% CI)**	***I*^2^ (%)**	**Spe (95% CI)**	***I*^2^ (%)**
SVM	16	0.90 [0.83∼0.94]	88.7	0.94 [0.84∼0.98]	94.5	20	0.85 [0.79∼0.90]	59.0	0.90 [0.84∼0.94]	66.9
LR	11	0.88 [0.70∼0.96]	97.1	0.88 [0.72∼0.95]	96.5	12	0.81 [0.70∼0.89]	85.6	0.84 [0.71∼0.92]	85.7
RF	5	0.97 [0.70∼1.00]	98.2	0.97 [0.54∼1.00]	98.8	11	0.79 [0.69∼0.87]	71.6	0.81 [0.68∼0.90]	77.0
LASSO	3	0.81∼0.94	NA	0.75∼0.96	NA	5	0.91 [0.77∼0.97]	67.0	0.90 [0.58∼0.98]	82.5
ANN	2	1.00	NA	1.00	NA	2	1.00	NA	1.00	NA
CNN	2	0.80∼0.86	NA	0.83∼0.88	NA	3	0.56∼0.86	NA	0.67∼0.70	NA
KNN	1	0.74	NA	0.74	NA	1	0.55	NA	0.64	NA
DT	1	0.69	NA	0.92	NA	1	0.59	NA	0.92	NA
Bayes	1	0.76	NA	0.92	NA	1	0.77	NA	0.96	NA
LDA	NA	NA	NA	NA	NA	4	0.97 [0.86∼1.00]	93.7	0.92 [0.80∼0.97]	23.3
Overall	42	0.91 [0.86∼0.94]	95.4	0.93 [0.87∼0.96]	95.8	60	0.86 [0.81∼0.89]	81.4	0.87 [0.83∼0.91]	82.0

When the number of models is less than four, it is not possible to perform a meta-analysis using a bivariate mixed-effects model. Therefore, we only recorded the corresponding exact values and range. Number: the number of models included in various model types. SVM, support vector machine; LR, logistic regression; RF, random forest; LASSO, least absolute shrinkage and selection operator; ANN, artificial neural network; CNN, convolutional neural network; KNN, K-nearest neighbor; DT, decision tree; Bayes, Bayesian network; LDA, linear discriminant analysis.

Among all the ML models constructed, support vector machine (SVM) and logistic regression (LR) showed ideal predictive performance in the training and validation sets with a larger sample size. Meanwhile, attention should also be paid to other models, such as CNN and LASSO, which demonstrated good diagnostic performance, despite a limited number of these models included in this study. Including more models in future studies can help verify their diagnostic potential.

#### 3.4.2. Differential diagnosis PD and APS

**FIGURE 4:**
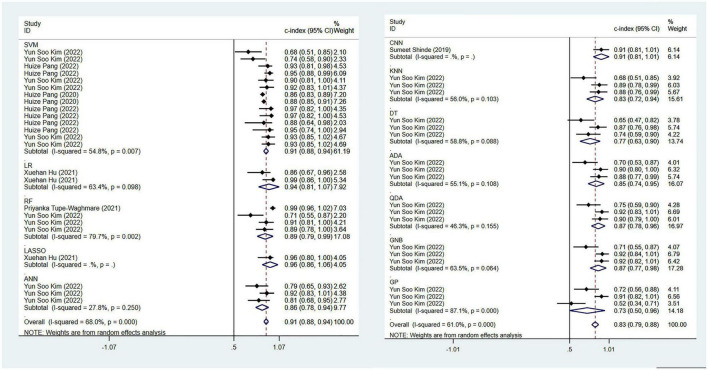
Meta-analysis results of c-index for differential diagnosis between PD and APS based on radiomics-based machine learning (Validation set).

Regarding the differential diagnosis between PD and APS, a total of 41 ML models reported a c-index, with a pooled c-index of 0.866 (95% CI: 0.843–0.889) in the training set, while in the validation set, 43 ML models reported a c-index, with a pooled c-index of 0.879 (95% CI: 0.854–0.903). The training set of 41 models had a pooled sensitivity and specificity of 0.86 (95% CI: 0.84–0.88) and 0.80 (95% CI: 0.75–0.84), respectively. Conversely, the validation set had a pooled sensitivity and specificity of 0.87 (95% CI: 0.85–0.89) and 0.82 (95% CI: 0.77–0.86), respectively. These results are detailed in Figure 4, Table 3 and Supplementary Table 6. Notably, the SVM model showed good discrimination accuracy even with a relatively large number of models included in the analysis.

**TABLE 3:** Meta-analysis results of sensitivity and specificity for differential diagnosis between PD and APS based on radiomics-based machine learning.

Model	Training set					Validation set				
	**Number**	**Sen (95% CI)**	***I*^2^ (%)**	**Spe (95% CI)**	***I*^2^ (%)**	**Number**	**Sen (95% CI)**	***I*^2^ (%)**	**Spe (95% CI)**	***I*^2^ (%)**
SVM	10	0.88 [0.82∼0.92]	62.5	0.83 [0.75∼0.89]	69.7	10	0.86 [0.80∼0.90]	0.0	0.84 [0.77∼0.89]	0.0
LR	2	0.76∼0.93	NA	0.91∼1.00	NA	2	0.89∼0.94	NA	0.78∼1.00	NA
RF	3	0.81∼0.88	NA	0.63∼0.89	NA	4	0.84 [0.73∼0.92]	0.0	0.86 [0.61∼0.96]	54.7
LASSO	1	0.91	NA	0.95	NA	1	0.83	NA	1.00	NA
ANN	3	0.77∼0.81	NA	0.51∼0.84	NA	3	0.79∼0.88	NA	0.59∼0.91	NA
CNN	4	0.90 [0.83∼0.95]	0.0	0.88 [0.71∼0.95]	91.0	3	0.91∼1.00	NA	0.50∼0.90	NA
KNN	3	0.81∼0.90	NA	0.49∼0.87	NA	3	0.79∼0.90	NA	0.56∼0.83	NA
DT	3	0.81∼0.88	NA	0.56∼0.84	NA	3	0.80∼0.88	NA	0.51∼0.82	NA
ADA	3	0.80∼0.89	NA	0.49∼0.82	NA	3	0.79∼0.88	NA	0.54∼0.80	NA
QDA	3	0.81∼0.91	NA	0.60∼0.85	NA	3	0.81∼0.91	NA	0.63∼0.85	NA
GNB	3	0.82∼0.92	NA	0.54∼0.89	NA	3	0.80∼0.94	NA	0.57∼0.87	NA
GP	3	0.78∼0.88	NA	0.66∼0.90	NA	3	0.78∼0.90	NA	0.74∼0.91	NA
Overall	41	0.86 [0.84∼0.88]	21.8	0.80 [0.75∼0.84]	75.1	41	0.87 [0.85∼0.89]	0.0	0.82 [0.77∼0.86]	18.4

ADA, adaptive boosting; QDA, quadratic discriminant analysis; GNB, Gaussian naive Bayes; GP, Gaussian process.

#### 3.4.3. Differential diagnosis of PD and MSA

**FIGURE 5:**
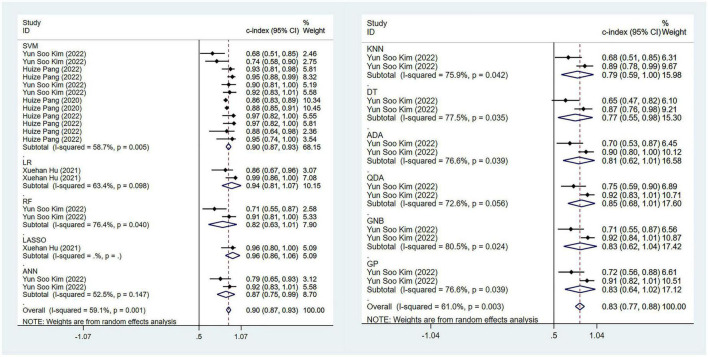
Meta-analysis results of c-index for differential diagnosis between PD and MSA based on radiomics-based machine learning (Validation set).

The pooled c-index, sensitivity, and specificity for differential diagnosis between PD and MSA were 0.857 (95% CI: 0.827–0.887), 0.86 (95% CI: 0.83–0.88), and 0.82 (95% CI: 0.77–0.87) in the training set, which contained 27 models, respectively. In the validation set, which included 31 models, the pooled c-index, sensitivity, and specificity were 0.878 (95% CI: 0.852–0.905), 0.85 (95% CI: 0.82–0.88), and 0.82 (95% CI: 0.77–0.87), respectively. These results are presented in Figure 5, Table 4 and Supplementary Table 7.

**TABLE 4:** Meta-analysis results of sensitivity and specificity for differential diagnosis between PD and MSA based on radiomics-based machine learning.

Model	Training set					Validation set				
	**Number**	**Sen (95% CI)**	***I*^2^ (%)**	**Spe (95% CI)**	***I*^2^ (%)**	**Number**	**Sen (95% CI)**	***I*^2^ (%)**	**Spe (95% CI)**	***I*^2^ (%)**
SVM	8	0.89 [0.81∼0.93]	66.5	0.86 [0.81∼0.90]	0.0	8	0.86 [0.79∼0.90]	0.0	0.84 [0.76∼0.90]	0.0
LR	2	0.76∼0.93	NA	0.91∼1.00	NA	2	0.89∼0.94	NA	0.78∼1.00	NA
RF	2	0.81∼0.88	NA	0.63∼0.89	NA	2	0.79∼0.88	NA	0.68∼0.90	NA
LASSO	1	0.91	NA	0.95	NA	1	0.83	NA	1.00	NA
ANN	2	0.77∼0.81	NA	0.73∼0.84	NA	2	0.79∼0.88	NA	0.75∼0.91	NA
KNN	2	0.81∼0.89	NA	0.49∼0.87	NA	2	0.79∼0.89	NA	0.56∼0.83	NA
DT	2	0.81∼0.86	NA	0.56∼0.84	NA	2	0.80∼0.87	NA	0.51∼0.82	NA
ADA	2	0.80∼0.88	NA	0.49∼0.82	NA	2	0.79∼0.88	NA	0.54∼0.80	NA
QDA	2	0.81∼0.91	NA	0.60∼0.85	NA	2	0.81∼0.91	NA	0.63∼0.85	NA
GNB	2	0.82∼0.90	NA	0.54∼0.89	NA	2	0.80∼0.91	NA	0.63∼0.87	NA
GP	2	0.78∼0.88	NA	0.79∼0.90	NA	2	0.78∼0.90	NA	0.82∼0.91	NA
Overall	27	0.86 [0.83∼0.88]	32.1	0.82 [0.77∼0.87]	63.9	27	0.85 [0.82∼0.88]	0.0	0.82 [0.77∼0.87]	2.0

#### 3.4.4. Differential diagnosis between PD and PSP

**FIGURE 6:**
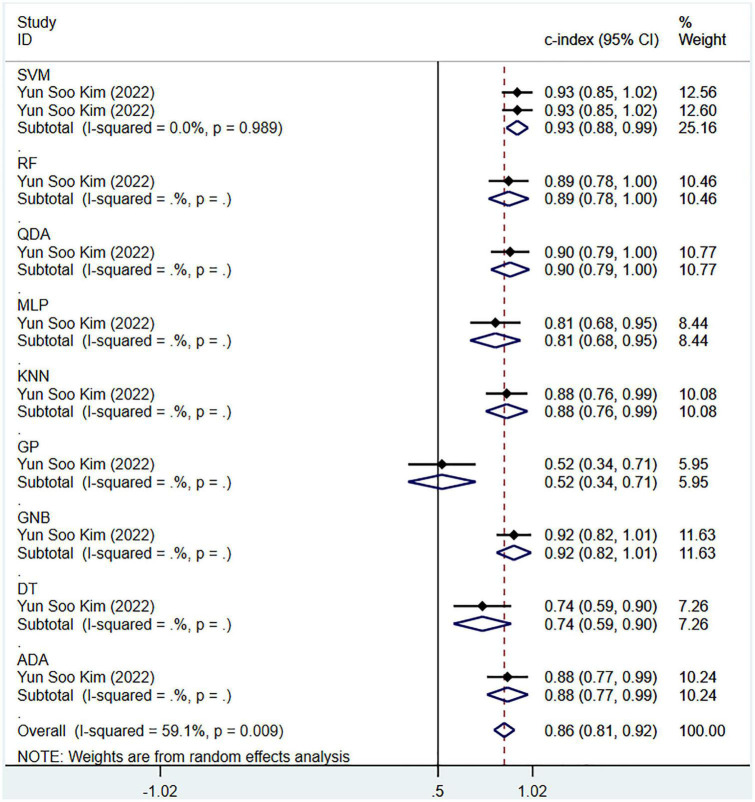
Meta-analysis results of c-index for differential diagnosis between PD and PSP based on radiomics-based machine learning (Validation set).

The pooled c-index, sensitivity, and specificity for differential diagnosis between PD and PSP in the training set of 10 models were 0.871 (95% CI: 0.826–0.915), 0.87 (95% CI: 0.82–0.90), and 0.63 (95% CI: 0.53–0.71), respectively. In the validation set, the pooled c-index, sensitivity, and specificity were 0.863 (95% CI: 0.808–0.918), 0.88 (95% CI: 0.82–0.92), and 0.68 (95% CI: 0.54–0.79), respectively. These findings are presented in Figure 6, Table 5 and Supplementary Table 8.

**TABLE 5:** Meta-analysis results of sensitivity and specificity for differential diagnosis between PD and PSP based on radiomics-based machine learning.

Model	Training set					Validation set				
	**Number**	**Sen (95% CI)**	***I*^2^ (%)**	**Spe (95% CI)**	***I*^2^ (%)**	**Number**	**Sen (95% CI)**	***I*^2^ (%)**	**Spe (95% CI)**	***I*^2^ (%)**
SVM	2	0.79∼0.91	NA	0.37∼0.75	NA	2	0.82∼0.91	NA	0.75∼0.79	NA
RF	1	0.87	NA	0.69	NA	1	0.88	NA	0.69	NA
ANN	1	0.80	NA	0.51	NA	1	0.80	NA	0.59	NA
KNN	1	0.90	NA	0.76	NA	1	0.90	NA	0.72	NA
DT	1	0.88	NA	0.67	NA	1	0.88	NA	0.60	NA
ADA	1	0.89	NA	0.65	NA	1	0.87	NA	0.62	NA
QDA	1	0.87	NA	0.71	NA	1	0.89	NA	0.68	NA
GNB	1	0.92	NA	0.59	NA	1	0.94	NA	0.57	NA
GP	1	0.79	NA	0.66	NA	1	0.81	NA	0.74	NA
Overall	10	0.87 [0.82∼0.90]	0.0	0.63 [0.53∼0.71]	0.0	10	0.88 [0.82∼0.92]	0.0	0.68 [0.54∼0.79]	0.0

#### 3.4.5. Differential diagnosis between different motor subtypes of PD

Regarding the differential diagnosis between TD and PIGD motor subtypes, there were three models in the training set and validation set, respectively. The pooled c-index was 0.892 (95% CI: 0.855–0.929) in the training set and 0.822 (95% CI: 0.724–0.920) in the validation set. The pooled sensitivity and specificity for TD subtype were between 0.85–0.88 and 0.77–0.82, respectively. For PIGD subtype, the pooled sensitivity and specificity were between 0.75–0.88 and 0.66–0.83, respectively. These results are presented in Supplementary Tables 9, 10.

### 3.5. Overfitting evaluation

For the diagnosis and differential diagnosis of PD, no overfitting was observed for the ML models. Meanwhile, in the respective differential diagnoses, no overfitting was observed for the most commonly used ML model when there were relatively sufficient models. The detailed information is shown in Supplementary Tables 5–9.

## 4. Discussion

Our meta-analysis results indicated that radiomics demonstrated excellent diagnostic accuracy in PD diagnosis, with a pooled sensitivity and specificity of 0.91 and 0.93 in the training set, and 0.86 and 0.87 in the validation set, respectively. Furthermore, radiomics-based ML has good discrimination performance in differentiating PD from APS and classifying PD subtypes.

In recent years, researchers have made significant progress in exploring biomarkers for the diagnosis of Parkinson’s disease (PD) (Parkinson Progression Marker Initiative, 2011; Tolosa et al., 2021). A meta-analysis of ML based on blood gene features for the prediction of idiopathic PD exhibited a sensitivity of 0.72 and specificity of 0.67 (Falchetti et al., 2020). Kalyakulina et al. (2022) conducted a meta-analysis of ML based on DNA methylation for the differentiation between PD cases and controls, with a classification accuracy of 0.76 using uncoordinated data and over 0.95 using coordinated data. di Biase et al. (2020) review reported an accuracy of over 0.83 for PD diagnosis using ML based on gait feature testing. Kwon et al. (2022) review demonstrated that the integration of clinically relevant biomarkers such as metabolomics, proteomics, and microRNA omics data from cerebrospinal fluid can serve as a powerful method for identifying PD and MSA. The aforementioned research results demonstrate that diagnostic models based on different variables have good performance in PD diagnosis. However, there have been no studies on the evaluation or integration of radiomics. Furthermore, the differentiation of PD and atypical parkinsonian syndromes (APS), as well as the classification of PD subtypes is rarely discussed. Previous studies have used conventional neuroimaging methods such as PET (Brajkovic et al., 2017), MRI, and molecular imaging (Atkinson-Clement et al., 2017; Loftus et al., 2023) for PD diagnosis based on visual assessment or statistical parameter mapping (SPM) analysis. Despite their high diagnostic accuracy, combining radiomics with artificial intelligence can save time and energy, reduce examination costs, and even improve diagnostic accuracy (Wu et al., 2019).

Previous studies have demonstrated that clinical factors, such as olfactory function (Alonso et al., 2021a,b), speech features, motor data, handwriting patterns, cardiac scintigraphy, cerebrospinal fluid (CSF), and serum markers, are closely associated with the diagnosis and severity assessment of Parkinson’s disease (PD) and should not be disregarded when constructing diagnostic models (Mei et al., 2021; Rana et al., 2022). Halligan et al. (2021) have recommended that multivariable models should include clinical imaging biomarkers to evaluate their cumulative contribution to overall outcomes. A review by Zhang (2022) has shown that multimodal data, based on ML using imaging and clinical features, can enhance the accuracy of PD diagnosis and early detection. Additionally, Makarious et al. (2022) have demonstrated in their review that multimodal data-combined ML models is superior to single biomarker mode, and the model has been validated in the PD Biomarker Program (PDBP) dataset. The ten studies included in this meta-analysis (Cao et al., 2020, 2021; Pang et al., 2020, 2022; Shu et al., 2020; Hu et al., 2021; Li et al., 2021; Zhang et al., 2021; Sun et al., 2022; Zhao et al., 2022) also revealed that comprehensive classification models, which combine clinical features and radiomics, have better predictive performance. Therefore, future radiomics analysis should incorporate other relevant variables to build more reliable models, and radiomic features can be added to existing diagnostic models to improve their diagnostic accuracy.

This study is the first systematic review and meta-analysis of radiomics-based ML in the diagnosis of PD and the differentiation of PD from APS. This study revealed that the main brain regions commonly used for diagnosis of PD were located in the substantia nigra-corpus striatum system, and some related areas such as the cerebral cortex. This was consistent with the pathological mechanism and features of PD. Some non-motor symptoms (olfactory disorder, depression, cognitive impairment, etc.) as non-radiomics variables for ML models had good value in diagnosing PD. Furthermore, we found that the major brain regions currently and commonly used to differentiate PD from APS were located in the basal ganglia system, especially the putamen area. UPDRS scores, as non-radiomics variables for ML model, were of good value in distinguishing PD from APD. The radiomics features commonly used to build ML models include first-order properties, shape features, and textural features [such as Gray Level Co-occurrence Matrix (GLCM), Gray Level Difference Matrix (GLDM), Gray-Level Run-Length Matrix (GLRLM)], etc.

We attempted to categorize models by type to determine the best model, but the number of some models, such as CNN, is limited due to their recent emergence, newer technology in deep learning (DL), and possible biases (Ching et al., 2018; Choi et al., 2020). DL has demonstrated greater potential for super-large datasets containing thousands or millions of cases (Camacho et al., 2018), whereas research datasets typically contain only hundreds of patients, making ML more suitable and cost-effective for building models for research purposes (Zhang et al., 2022). In our study, DL also demonstrated good diagnostic prediction performance, but we cannot draw definitive conclusions due to the limited number of the included studies. Further research is needed to endorse these findings. However, the SVM model still demonstrates excellent robustness even when the number of samples is relatively abundant. Additionally, we found that MRI was the main tool that used radiomics to predict PD diagnosis in clinical practice. In future work, incorporating data from various imaging modalities can further enhance the diagnostic capabilities for the disease. Our findings may advance the field of digital therapy and provide theoretical evidence for developing ML models for diagnosing PD in the future.

However, this study has certain limitations. Firstly, Currently, radiomics lacks a standardized operational guideline, which leads to variations in the process of region of interest (ROI) delineation and texture feature extraction among researchers. Even when multiple researchers are involved, it appears challenging to eliminate the impact of these variations. Additionally, the use of diverse dimensionality reduction methods or variable selection methods may contribute to high heterogeneity in radiomics studies targeting the same clinical question. Therefore, these factors may introduce a significant heterogeneity in systematic reviews related to radiomics. It is difficult to avoid such heterogeneity until standardized operational guidelines are widely adopted. Secondly, we observed that the included studies seemed to have relatively low scores, mainly due to the fact that the RQS scale is more inclined toward critical research on radiomics. Additionally, the RQS scale may be unsuitable for some models in clinical practice, making it difficult for some studies to obtain high RQS scores. Moreover, many related studies currently have a retrospective design, are single-center studies, and use internal validation or resampling methods (cross-validation), resulting in poor generalizability of the models and limiting the integration of ML models with clinical environments. Therefore, in the future, images from different hospitals and research centers are needed to externally validate the prediction model, making it adapt to a wider range of clinical scenarios. Furthermore, not all models are suitable for clinical practice, so the clinical effectiveness of diagnostic models must be strictly evaluated based on current diagnostic standards.

Imaging plays an indispensable role in the clinical diagnosis and treatment process. However, the interpretation of imaging data currently relies primarily on the expertise of clinical experts. In this regard, developing an intelligent radiomics reading tool based on standardized criteria would provide significant assistance to novice clinicians, especially in the diagnosis and treatment of complex diseases. This assistance in radiomics-based interpretation is crucial for clinical practice. Furthermore, promoting the development of radiomics can bring substantial value to the initial screening and diagnosis of many diseases, particularly in economically and medically underdeveloped regions.

However, radiomics currently faces several inevitable challenges and problems, with significant biases present in certain aspects of the radiomics implementation process. The development of radiomics did not adequately consider excessive parameter tuning, nor did it involve repeated measurements at different time points on the same patient (although this incurs certain costs, it is necessary for the development of such a tool). Moreover, the delineation of the ROI heavily relies on the expertise and knowledge of clinical experts. Therefore, in the development process, it is essential to incorporate ROI delineation from clinicians at different levels to generate imaging data, followed by the extraction of radiomics features using specific software. We have observed strong correlations among some of the extracted radiomics variables, making the selection of modeling variables a challenging task. Hence, it is crucial to compare different methods and identify the optimal variable selection approach to build ML models while avoiding overfitting. Additionally, in the process of constructing ML models, it may be advantageous to prioritize logistic regression (LR) as it offers good visualization and relatively straightforward predictive line plots. We hope that better standards for radiomics and ML will be established in the future, such as the standardization of image acquisition, segmentation, feature extraction, statistical analysis, and reporting formats, to achieve reproducibility and facilitate clinical application.

## 5. Conclusion

Our study suggested that radiomic-based ML exhibited high sensitivity and specificity in diagnosing Parkinson’s disease (PD), discriminating PD and atypical parkinsonian syndromes (APS), and distinguishing different subtypes of PD. This approach can serve as a potential method for screening, detecting, and diagnosing PD, making a significant contribution to clinical decision-making systems. However, due to the current lack of standardized operational guidelines, radiomics still faces numerous challenges in its current applications.

## Data availability statement

The original contributions presented in this study are included in the article/Supplementary material, further inquiries can be directed to the corresponding author.

## Author contributions

JB and XW: conceptualization. XW: resources. XW and WH: methodology. JB: formal analysis and investigation. JB and WH: writing—original draft preparation. GZ: writing—review and editing. YW: supervision. All authors read and approved the final manuscript.
