# Supplementary material for: The differential diagnosis value of radiomics-based machine learning in Parkinson’s disease: a systematic review and meta-analysis
Source: Front Aging Neurosci. 2023 Jul 6;15:1199826. doi: 10.3389/fnagi.2023.1199826 (PMC10357514; doi:10.3389/fnagi.2023.1199826)
Supplement: Supplementary file 1 [file Data_Sheet_1.docx]

Supplementary Material

The differential diagnosis value of radiomics-based machine learning in Parkinson's disease: a systematic review and meta-analysis

Jiaxiang Bian, Xiaoyang Wang, Wei Hao, Guangjian Zhang, Yuting Wang^*^

*** Correspondence:** Yuting Wang: 15253669990@163.com

# Supplementary Tables

**Supplementary Table S1.** PRISMA 2020 Checklist.

| **Section and Topic** | **Item #** | **Checklist item** | **Location where item is reported** |
| --- | --- | --- | --- |
| **TITLE** | | |  |
| Title | 1 | Identify the report as a systematic review. | Page 1 |
| **ABSTRACT** | | |  |
| Abstract | 2 | See the PRISMA 2020 for Abstracts checklist. | Page 1-2/Line 10-34 |
| **INTRODUCTION** | | |  |
| Rationale | 3 | Describe the rationale for the review in the context of existing knowledge. | Page 2/Line 36-64 |
| Objectives | 4 | Provide an explicit statement of the objective(s) or question(s) the review addresses. | Page 2/Line 64-65 |
| **METHODS** | | |  |
| Eligibility criteria | 5 | Specify the inclusion and exclusion criteria for the review and how studies were grouped for the syntheses. | Page 2-3/Line 71-83 |
| Information sources | 6 | Specify all databases, registers, websites, organisations, reference lists and other sources searched or consulted to identify studies. Specify the date when each source was last searched or consulted. | Page 3/Line 85-88 |
| Search strategy | 7 | Present the full search strategies for all databases, registers and websites, including any filters and limits used. | Page 3/Line 85-88 |
| Selection process | 8 | Specify the methods used to decide whether a study met the inclusion criteria of the review, including how many reviewers screened each record and each report retrieved, whether they worked independently, and if applicable, details of automation tools used in the process. | Page 2-3/Line 71-106 |
| Data collection process | 9 | Specify the methods used to collect data from reports, including how many reviewers collected data from each report, whether they worked independently, any processes for obtaining or confirming data from study investigators, and if applicable, details of automation tools used in the process. | Page 3/Line 90-106 |
| Data items | 10a | List and define all outcomes for which data were sought. Specify whether all results that were compatible with each outcome domain in each study were sought (e.g. for all measures, time points, analyses), and if not, the methods used to decide which results to collect. | Page 3/Line 90-106 |
|  | 10b | List and define all other variables for which data were sought (e.g. participant and intervention characteristics, funding sources). Describe any assumptions made about any missing or unclear information. | Page 3/Line 90-106 |
| Study risk of bias assessment | 11 | Specify the methods used to assess risk of bias in the included studies, including details of the tool(s) used, how many reviewers assessed each study and whether they worked independently, and if applicable, details of automation tools used in the process. | Page 3-4/Line 108-118 |
| Effect measures | 12 | Specify for each outcome the effect measure(s) (e.g. risk ratio, mean difference) used in the synthesis or presentation of results. | Page 4/Line 120-124 |
| Synthesis methods | 13a | Describe the processes used to decide which studies were eligible for each synthesis (e.g. tabulating the study intervention characteristics and comparing against the planned groups for each synthesis (item #5)). | Page 4/Line 126-144 |
|  | 13b | Describe any methods required to prepare the data for presentation or synthesis, such as handling of missing summary statistics, or data conversions. | Page 4/Line 126-144 |
|  | 13c | Describe any methods used to tabulate or visually display results of individual studies and syntheses. | Page 4/Line 126-144 |
|  | 13d | Describe any methods used to synthesize results and provide a rationale for the choice(s). If meta-analysis was performed, describe the model(s), method(s) to identify the presence and extent of statistical heterogeneity, and software package(s) used. | Page 4/Line 126-144 |
|  | 13e | Describe any methods used to explore possible causes of heterogeneity among study results (e.g. subgroup analysis, meta-regression). | Page 4/Line 126-144 |
|  | 13f | Describe any sensitivity analyses conducted to assess robustness of the synthesized results. | Page 4/Line 126-144 |
| Reporting bias assessment | 14 | Describe any methods used to assess risk of bias due to missing results in a synthesis (arising from reporting biases). | Page 3-4/Line 108-144 |
| Certainty assessment | 15 | Describe any methods used to assess certainty (or confidence) in the body of evidence for an outcome. | Page 3-4/Line 108-144 |
| **RESULTS** | | |  |
| Study selection | 16a | Describe the results of the search and selection process, from the number of records identified in the search to the number of studies included in the review, ideally using a flow diagram. | Page 4/Line 147-151 |
|  | 16b | Cite studies that might appear to meet the inclusion criteria, but which were excluded, and explain why they were excluded. | Page 4/Line 147-151 |
| Study characteristics | 17 | Cite each included study and present its characteristics. | Page 5/Line 153-165 |
| Risk of bias in studies | 18 | Present assessments of risk of bias for each included study. | Page 5/Line 167-190 |
| Results of individual studies | 19 | For all outcomes, present, for each study: (a) summary statistics for each group (where appropriate) and (b) an effect estimate and its precision (e.g. confidence/credible interval), ideally using structured tables or plots. | Page 5-7/Line 192-244 |
| Results of syntheses | 20a | For each synthesis, briefly summarise the characteristics and risk of bias among contributing studies. | Page 5-7/Line 192-244 |
|  | 20b | Present results of all statistical syntheses conducted. If meta-analysis was done, present for each the summary estimate and its precision (e.g. confidence/credible interval) and measures of statistical heterogeneity. If comparing groups, describe the direction of the effect. | Page 5-7/Line 192-244 |
|  | 20c | Present results of all investigations of possible causes of heterogeneity among study results. | Page 5-7/Line 192-244 |
|  | 20d | Present results of all sensitivity analyses conducted to assess the robustness of the synthesized results. | Page 5-7/Line 192-244 |
| Reporting biases | 21 | Present assessments of risk of bias due to missing results (arising from reporting biases) for each synthesis assessed. | Page 5-7/Line 67-244 |
| Certainty of evidence | 22 | Present assessments of certainty (or confidence) in the body of evidence for each outcome assessed. | Page 5-7/Line 167-244 |
| **DISCUSSION** | | |  |
| Discussion | 23a | Provide a general interpretation of the results in the context of other evidence. | Page 7-8/Line 246-296 |
|  | 23b | Discuss any limitations of the evidence included in the review. | Page 8/Line 297-312 |
|  | 23c | Discuss any limitations of the review processes used. | Page 8/Line 297-312 |
|  | 23d | Discuss implications of the results for practice, policy, and future research. | Page 7-8/Line 246-296 |
| **OTHER INFORMATION** | | |  |
| Registration and protocol | 24a | Provide registration information for the review, including register name and registration number, or state that the review was not registered. | Page 2/Line 67-69 |
|  | 24b | Indicate where the review protocol can be accessed, or state that a protocol was not prepared. | Page 2/Line 67-69 |
|  | 24c | Describe and explain any amendments to information provided at registration or in the protocol. | Page 2/Line 67-69 |
| Support | 25 | Describe sources of financial or non-financial support for the review, and the role of the funders or sponsors in the review. | Page 9/Line 327 |
| Competing interests | 26 | Declare any competing interests of review authors. | Page 8/Line 319-320 |
| Availability of data, code and other materials | 27 | Report which of the following are publicly available and where they can be found: template data collection forms; data extracted from included studies; data used for all analyses; analytic code; any other materials used in the review. | Page 14/Line 517-518 |

*From:*  Page MJ, McKenzie JE, Bossuyt PM, Boutron I, Hoffmann TC, Mulrow CD, et al. The PRISMA 2020 statement: an updated guideline for reporting systematic reviews. BMJ 2021;372:n71. doi: 10.1136/bmj.n71

For more information, visit: <http://www.prisma-statement.org/>

**Supplementary Table S2.** Literature search strategy.

**1.Pubmed**

| Search number | Query | Results |
| --- | --- | --- |
| #1 | "Parkinson Disease"[Mesh] | 78907 |
| #2 | ((((((((((((((((((((((((Parkinson Disease[Title/Abstract]) OR (Idiopathic Parkinson's Disease[Title/Abstract])) OR (Lewy Body Parkinson's Disease[Title/Abstract])) OR (Parkinson's Disease, Idiopathic[Title/Abstract])) OR (Parkinson's Disease, Lewy Body[Title/Abstract])) OR (Parkinson Disease, Idiopathic[Title/Abstract])) OR (Parkinson's Disease[Title/Abstract])) OR (Idiopathic Parkinson Disease[Title/Abstract])) OR (Lewy Body Parkinson Disease[Title/Abstract])) OR (Primary Parkinsonism[Title/Abstract])) OR (Parkinsonism, Primary[Title/Abstract])) OR (Paralysis Agitans[Title/Abstract])) OR (Parkinsons disease[Title/Abstract])) OR (idiopathic parkinsonism[Title/Abstract])) OR (Lewy bodies of Parkinson disease[Title/Abstract])) OR (Lewy bodies of Parkinson's disease[Title/Abstract])) OR (Lewy bodies of Parkinsons disease[Title/Abstract])) OR (Lewy body Parkinson disease[Title/Abstract])) OR (Lewy body Parkinson's disease[Title/Abstract])) OR (Lewy body Parkinsons disease[Title/Abstract])) OR (paralysis agitans[Title/Abstract])) OR (Parkinson dementia complex[Title/Abstract])) OR (Parkinson's disease[Title/Abstract])) OR (Parkinsons disease[Title/Abstract])) OR (primary parkinsonism[Title/Abstract]) | 113402 |
| #3 | ("Parkinson Disease"[Mesh]) OR (((((((((((((((((((((((((Parkinson Disease[Title/Abstract]) OR (Idiopathic Parkinson's Disease[Title/Abstract])) OR (Lewy Body Parkinson's Disease[Title/Abstract])) OR (Parkinson's Disease, Idiopathic[Title/Abstract])) OR (Parkinson's Disease, Lewy Body[Title/Abstract])) OR (Parkinson Disease, Idiopathic[Title/Abstract])) OR (Parkinson's Disease[Title/Abstract])) OR (Idiopathic Parkinson Disease[Title/Abstract])) OR (Lewy Body Parkinson Disease[Title/Abstract])) OR (Primary Parkinsonism[Title/Abstract])) OR (Parkinsonism, Primary[Title/Abstract])) OR (Paralysis Agitans[Title/Abstract])) OR (Parkinsons disease[Title/Abstract])) OR (idiopathic parkinsonism[Title/Abstract])) OR (Lewy bodies of Parkinson disease[Title/Abstract])) OR (Lewy bodies of Parkinson's disease[Title/Abstract])) OR (Lewy bodies of Parkinsons disease[Title/Abstract])) OR (Lewy body Parkinson disease[Title/Abstract])) OR (Lewy body Parkinson's disease[Title/Abstract])) OR (Lewy body Parkinsons disease[Title/Abstract])) OR (paralysis agitans[Title/Abstract])) OR (Parkinson dementia complex[Title/Abstract])) OR (Parkinson's disease[Title/Abstract])) OR (Parkinsons disease[Title/Abstract])) OR (primary parkinsonism[Title/Abstract])) | 126218 |
| #4 | (((((Radiomics[Title/Abstract]) OR (radiomic[Title/Abstract])) OR (radiogenomic[Title/Abstract])) OR (radiomics-based[Title/Abstract])) OR (radiomic signature[Title/Abstract])) OR (Radiography[Title/Abstract]) | 79870 |
| #5 | (("Parkinson Disease"[Mesh]) OR (((((((((((((((((((((((((Parkinson Disease[Title/Abstract]) OR (Idiopathic Parkinson's Disease[Title/Abstract])) OR (Lewy Body Parkinson's Disease[Title/Abstract])) OR (Parkinson's Disease, Idiopathic[Title/Abstract])) OR (Parkinson's Disease, Lewy Body[Title/Abstract])) OR (Parkinson Disease, Idiopathic[Title/Abstract])) OR (Parkinson's Disease[Title/Abstract])) OR (Idiopathic Parkinson Disease[Title/Abstract])) OR (Lewy Body Parkinson Disease[Title/Abstract])) OR (Primary Parkinsonism[Title/Abstract])) OR (Parkinsonism, Primary[Title/Abstract])) OR (Paralysis Agitans[Title/Abstract])) OR (Parkinsons disease[Title/Abstract])) OR (idiopathic parkinsonism[Title/Abstract])) OR (Lewy bodies of Parkinson disease[Title/Abstract])) OR (Lewy bodies of Parkinson's disease[Title/Abstract])) OR (Lewy bodies of Parkinsons disease[Title/Abstract])) OR (Lewy body Parkinson disease[Title/Abstract])) OR (Lewy body Parkinson's disease[Title/Abstract])) OR (Lewy body Parkinsons disease[Title/Abstract])) OR (paralysis agitans[Title/Abstract])) OR (Parkinson dementia complex[Title/Abstract])) OR (Parkinson's disease[Title/Abstract])) OR (Parkinsons disease[Title/Abstract])) OR (primary parkinsonism[Title/Abstract]))) AND ((((((Radiomics[Title/Abstract]) OR (radiomic[Title/Abstract])) OR (radiogenomic[Title/Abstract])) OR (radiomics-based[Title/Abstract])) OR (radiomic signature[Title/Abstract])) OR (Radiography[Title/Abstract])) | 67 |

**2.Cochrane**

| Search number | Query | Results |
| --- | --- | --- |
| #1 | MeSH descriptor: [Parkinson Disease] explode all trees | 4814 |
| #2 | (Parkinson Disease):ti,ab,kw OR (Idiopathic Parkinson's Disease):ti,ab,kw OR (Lewy Body Parkinson's Disease):ti,ab,kw OR (Parkinson's Disease, Idiopathic):ti,ab,kw OR (Parkinson's Disease, Lewy Body):ti,ab,kw | 11498 |
| #3 | (Parkinson Disease, Idiopathic):ti,ab,kw OR (Parkinson's Disease):ti,ab,kw OR (Idiopathic Parkinson Disease):ti,ab,kw OR (Lewy Body Parkinson Disease):ti,ab,kw OR (Primary Parkinsonism):ti,ab,kw | 11631 |
| #4 | (Parkinsonism, Primary):ti,ab,kw OR (Paralysis Agitans):ti,ab,kw OR (Parkinsons disease):ti,ab,kw OR (idiopathic parkinsonism):ti,ab,kw OR (Lewy bodies of Parkinson disease):ti,ab,kw | 1441 |
| #5 | (Lewy bodies of Parkinson's disease):ti,ab,kw OR (Lewy bodies of Parkinsons disease):ti,ab,kw OR (Lewy body Parkinson disease):ti,ab,kw OR (Lewy body Parkinson's disease):ti,ab,kw OR (Lewy body Parkinsons disease):ti,ab,kw | 264 |
| #6 | (paralysis agitans):ti,ab,kw OR (Parkinson dementia complex):ti,ab,kw OR (Parkinson's disease):ti,ab,kw OR (Parkinsons disease):ti,ab,kw OR (primary parkinsonism):ti,ab,kw | 11687 |
| #7 | #1 or #2 or #3 or #4 or #5 or #6 | 11697 |
| #8 | (Radiomics):ti,ab,kw OR (radiomic):ti,ab,kw OR (radiogenomic):ti,ab,kw OR (radiomics-based):ti,ab,kw OR (radiomic signature):ti,ab,kw | 482 |
| #9 | (Radiography):ti,ab,kw | 11751 |
| #10 | #8 or #9 | 12229 |
| #11 | #7 and #10 | 14 |

**3.Embase**

| Search number | Query | Results |
| --- | --- | --- |
| #1 | 'parkinson disease'/exp | 185948 |
| #2 | 'parkinson disease'/exp OR 'parkinson disease' OR (parkinson AND ('disease'/exp OR disease)) OR 'idiopathic parkinsons disease':ab,ti OR 'parkinsons disease, idiopathic':ab,ti OR 'parkinsons disease, lewy body':ab,ti OR 'parkinson disease, idiopathic':ab,ti OR 'idiopathic parkinson disease':ab,ti OR 'parkinsonism, primary':ab,ti OR 'idiopathic parkinsonism':ab,ti OR 'lewy bodies of parkinson disease':ab,ti OR 'lewy bodies of parkinsons disease':ab,ti OR 'lewy body parkinson disease':ab,ti OR 'lewy body parkinsons disease':ab,ti OR 'paralysis agitans':ab,ti OR 'parkinson dementia complex':ab,ti OR 'parkinsons disease':ab,ti OR 'primary parkinsonism':ab,ti | 240207 |
| #3 | #1 OR #2 | 240207 |
| #4 | 'radiomics'/exp | 6151 |
| #5 | radiomics:ab,ti OR radiomic:ab,ti OR radiogenomic:ab,ti OR 'radiomics based':ab,ti OR 'radiomic signature':ab,ti OR radiography:ab,ti | 69314 |
| #6 | #4 OR #5 | 69896 |
| #7 | #3 AND #6 | 117 |

**4.Web of science**

| Search number | Query | Results |
| --- | --- | --- |
| #1 | Parkinson Disease (Topic) OR Idiopathic Parkinson's Disease (Topic) OR Lewy Body Parkinson's Disease (Topic) OR Parkinson's Disease, Idiopathic (Topic) OR Parkinson's Disease, Lewy Body (Topic) OR Parkinson Disease, Idiopathic (Topic) OR Parkinson's Disease (Topic) OR Idiopathic Parkinson Disease (Topic) OR Lewy Body Parkinson Disease (Topic) OR Primary Parkinsonism (Topic) OR Parkinsonism, Primary (Topic) OR Paralysis Agitans (Topic) OR Parkinsons disease (Topic) OR idiopathic parkinsonism (Topic) OR Lewy bodies of Parkinson disease (Topic) OR Lewy bodies of Parkinson's disease (Topic) OR Lewy bodies of Parkinsons disease (Topic) OR Lewy body Parkinson disease (Topic) OR Lewy body Parkinson's disease (Topic) OR Lewy body Parkinsons disease (Topic) OR paralysis agitans (Topic) OR Parkinson dementia complex (Topic) OR Parkinson's disease (Topic) OR Parkinsons disease (Topic) OR primary parkinsonism (Topic) | 183165 |
| #2 | Radiomics (Topic) OR radiomic (Topic) OR radiogenomic (Topic) OR radiomics-based (Topic) OR radiomic signature (Topic) OR Radiography (Topic) | 72387 |
| #3 | #2 AND #1 | 75 |

**Supplementary Table S3.** Quality evaluation score sheet.

| First author | Year | v1 | v2 | v3 | v4 | v5 | v6 | v7 | v8 | v9 | v10 | v11 | v12 | v13 | v14 | v15 | v16 | Total score | Relative score(%) |
| --- | --- | --- | --- | --- | --- | --- | --- | --- | --- | --- | --- | --- | --- | --- | --- | --- | --- | --- | --- |
| Yu Zhao | 2022 | 1 | 0 | 0 | 0 | 3 | 1 | 0 | 1 | 1 | 0 | 0 | 2 | 0 | 2 | 0 | 3 | 14 | 38.89 |
| Xiaoming Sun | 2022 | 1 | 0 | 0 | 0 | 3 | 1 | 0 | 0 | 1 | 0 | 0 | 3 | 0 | 0 | 0 | 2 | 11 | 30.56 |
| Takuro Shiib | 2022 | 1 | 0 | 0 | 0 | 3 | 0 | 0 | 1 | 1 | 0 | 0 | 2 | 0 | 0 | 0 | 1 | 9 | 25.00 |
| Dafa Shi | 2022 | 1 | 0 | 0 | 0 | 3 | 0 | 0 | 0 | 1 | 0 | 0 | 3 | 0 | 0 | 0 | 2 | 10 | 27.78 |
| Dafa Shi | 2022 | 1 | 0 | 0 | 0 | 3 | 0 | 0 | 0 | 1 | 0 | 0 | 4 | 0 | 0 | 0 | 2 | 11 | 30.56 |
| Huize Pang | 2022 | 1 | 0 | 0 | 0 | 3 | 1 | 0 | 0 | 1 | 0 | 0 | 2 | 0 | 0 | 0 | 1 | 9 | 25.00 |
| Jingwen Li | 2022 | 1 | 0 | 0 | 0 | 3 | 0 | 0 | 1 | 1 | 0 | 0 | 2 | 0 | 0 | 0 | 2 | 10 | 27.78 |
| Yun Soo Kim | 2022 | 1 | 0 | 0 | 0 | 3 | 0 | 0 | 0 | 1 | 0 | 0 | 2 | 0 | 0 | 0 | 2 | 9 | 25.00 |
| Jin Juan Kang | 2022 | 1 | 0 | 0 | 0 | 3 | 0 | 0 | 0 | 1 | 1 | 0 | 2 | 0 | 0 | 0 | 2 | 10 | 27.78 |
| Xiao-Jun Guan | 2022 | 1 | 0 | 0 | 0 | 3 | 0 | 0 | 1 | 1 | 0 | 0 | 2 | 0 | 0 | 0 | 2 | 10 | 27.78 |
| Dafna Ben Bashat | 2022 | 1 | 0 | 0 | 0 | 3 | 0 | 1 | 0 | 1 | 0 | 0 | 2 | 2 | 0 | 0 | 2 | 12 | 33.33 |
| Xulian Zhang | 2021 | 1 | 1 | 0 | 0 | 3 | 0 | 0 | 0 | 1 | 0 | 0 | 2 | 0 | 0 | 0 | 2 | 10 | 27.78 |
| Priyanka Tupe-Waghmare | 2021 | 1 | 0 | 0 | 0 | 3 | 0 | 0 | 0 | 1 | 0 | 0 | 2 | 0 | 0 | 0 | 2 | 9 | 25.00 |
| Dong Sun | 2021 | 1 | 0 | 0 | 0 | 3 | 0 | 0 | 0 | 1 | 1 | 0 | 2 | 0 | 0 | 0 | 2 | 10 | 27.78 |
| Dafa Shi | 2021 | 1 | 0 | 0 | 0 | 3 | 0 | 0 | 1 | 1 | 0 | 0 | 2 | 0 | 0 | 0 | 1 | 9 | 25.00 |
| Qingguo Ren | 2021 | 1 | 1 | 0 | 0 | 3 | 0 | 0 | 1 | 1 | 0 | 0 | 2 | 0 | 2 | 0 | 2 | 13 | 36.11 |
| Xue-ning Li | 2021 | 1 | 1 | 0 | 0 | 3 | 1 | 0 | 0 | 1 | 0 | 0 | 2 | 0 | 0 | 0 | 2 | 11 | 30.56 |
| Xuehan Hu | 2021 | 1 | 1 | 0 | 0 | 3 | 1 | 0 | 0 | 1 | 1 | 0 | 2 | 0 | 2 | 0 | 2 | 14 | 38.89 |
| Nikhil J. Dhinagar | 2021 | 1 | 0 | 0 | 0 | 3 | 0 | 0 | 0 | 1 | 0 | 0 | 3 | 0 | 0 | 0 | 2 | 10 | 27.78 |
| Xuan Cao | 2021 | 1 | 0 | 0 | 0 | 3 | 1 | 0 | 1 | 1 | 0 | 0 | 2 | 0 | 0 | 0 | 2 | 11 | 30.56 |
| Zhenyu Shu | 2020 | 1 | 1 | 0 | 0 | 3 | 1 | 0 | 1 | 1 | 1 | 0 | 2 | 0 | 2 | 0 | 2 | 15 | 41.67 |
| Huize Pang | 2020 | 1 | 1 | 0 | 0 | 3 | 1 | 0 | 0 | 1 | 0 | 0 | 2 | 0 | 0 | 0 | 2 | 11 | 30.56 |
| Panshi Liu | 2020 | 1 | 1 | 0 | 0 | 3 | 0 | 0 | 1 | 1 | 0 | 0 | 2 | 0 | 0 | 0 | 2 | 11 | 30.56 |
| Xuan Cao | 2020 | 1 | 1 | 0 | 0 | 3 | 0 | 0 | 0 | 1 | 0 | 0 | 2 | 0 | 0 | 0 | 2 | 10 | 27.78 |
| Bin Xiao | 2019 | 1 | 0 | 0 | 0 | 3 | 0 | 0 | 0 | 1 | 0 | 0 | 2 | 0 | 0 | 0 | 1 | 8 | 22.22 |
| Yue Wu | 2019 | 1 | 0 | 0 | 0 | 3 | 0 | 0 | 0 | 1 | 0 | 0 | 3 | 0 | 2 | 0 | 1 | 11 | 30.56 |
| Sumeet Shinde | 2019 | 1 | 1 | 0 | 0 | 3 | 0 | 0 | 0 | 1 | 0 | 0 | 2 | 0 | 0 | 0 | 1 | 9 | 25.00 |
| Zenghui Cheng | 2019 | 1 | 1 | 0 | 0 | 3 | 0 | 0 | 0 | 1 | 1 | 0 | 2 | 0 | 0 | 0 | 2 | 11 | 30.56 |

Note:

1. Relative score calculation formula: Relative score(%)=Total score/36*100%（Retain two decimal places）
2. V1: Image protocol quality - well-documented image protocols (for example, contrast, slice thickness, energy, etc.) and/or usage of public image protocols allow reproducibility/replicability.

V2: Multiple segmentations - possible actions are: segmentation by different physicians/algorithms/software, perturbing segmentations by (random) noise, segmentation at different breathing cycles. Analyse feature robustness to segmentation variabilities.

V3: Phantom study on all scanners - detect inter-scanner differences and vendor-dependent features. Analyse feature robustness to these sources of variability.

V4: Imaging at multiple time points - collect images of individuals at additional time points. Analyse feature robustness to temporal variabilities (for example, organ movement, organ expansion/ shrinkage)

V5: Feature reduction or adjustment for multiple testing - decreases the risk of overfitting. Overfitting is inevitable if the number of features exceeds the number of samples. Consider feature robustness when selecting features.

V6: Multivariable analysis with non radiomics features (for example, EGFR mutation) - is expected to provide a more holistic model. Permits correlating/inferencing between radiomics and non radiomics features.

V7: Detect and discuss biological correlates - demonstration of phenotypic differences (possibly associated with underlying gene–protein expression patterns) deepens understanding of radiomics and biology.

V8: Cut-off analyses - determine risk groups by either the median, a previously published cut-off or report a continuous risk variable. Reduces the risk of reporting overly optimistic results---Rscore.

V9: Discrimination statistics - report discrimination statistics (for example, C-statistic, ROC curve, AUC) and their statistical significance (for example, p-values, confidence intervals). One can also apply resampling method (for example, bootstrapping, cross-validation).

V10: Calibration statistics - report calibration statistics (for example, Calibration-in-the-large/slope, calibration plots) and their statistical significance (for example, P-values, confidence intervals). One can also apply resampling method (for example, bootstrapping, cross-validation).

V11: Prospective study registered in a trial database - provides the highest level of evidence supporting the clinical validity and usefulness of the radiomics biomarker.

V12: Validation - the validation is performed without retraining and without adaptation of the cut-off value, provides crucial information with regard to credible clinical performance.

V13: Comparison to ‘gold standard’ - assess the extent to which the model agrees with/is superior to the current ‘gold standard’ method (for example, TNM-staging for survival prediction). This comparison shows the added value of radiomics.

V14: Potential clinical utility - report on the current and potential application of the model in a clinical setting (for example, decision curve analysis).

V15: Cost-effectiveness analysis - report on the cost-effectiveness of the clinical application (for example, QALYs generated).

V16: Open science and data - make code and data publicly available. Open science facilitates knowledge transfer and reproducibility of the study.

**The following are the modifications and additions:**

**Supplementary Table S4.** Modeling information of the included studies.

| **No.** | **First author** | **Year** | **Related brain regions** | **Radiomics features** | **The imaging modality** | **Clinical biomarkers (Non-radiomic modeling variable)** |
| --- | --- | --- | --- | --- | --- | --- |
| 1 | Yu Zhao | 2022 | The putamen, caudate and deep learning-derived important regions | The deep-learning-guided radiomics features. | PET (DAT PET) | Age, gender, symptom duration, UPDRS, Hoehn and Yahr stage. |
| 2 | Xiaoming Sun | 2022 | SFG, MFG, SMA, OG, CN, PUT, GP, thalamus, ITG, cerebellum, and pons. | Texture features (Histogram-based texture features, GLCM features, GLRLM features, GLSZM features, NGTDM features),  Intensity features (SUVmax, SUVpeak, SUVmean, aucCSH) | PET (18F-FDG PET) | Age, sex |
| 3 | Takuro Shiib | 2022 | The caudate, putamen, and pallidum | Four texture features from the putamen: Intensity histogram (Median), GLDZM (Zone distance non-uniformity), two NGLDM (Dependence count non-uniformity normalised),  (GLDZM (Zone distance non-uniformity) resulted in the most significant coefficient.) One from caudate/pallidum: GLSZM (Large zone low grey-level emphasis) | SPECT (DAT SPECT) | NA |
| 4 | Dafa Shi | 2022 | The SMN and lateral parietal cortex (SFG, PrG, PCL_R, PCun, ITG_L, SPL) The brain regions were mainly located in the frontal lobe, especially SFG. | Intensity histogram-, texture-, and wavelet transformation-based features (first- and high-order features) | MRI (Rs-fMRI) | NA |
| 5 | Dafa Shi | 2022 | The right IFG, PhG, STG, left PoG, PrG, MFG, SFG | Intensity-based histogram features (minimum, kurtosis, tenth percentile, median) | MRI (Rs-fMRI) | NA |
| 6 | Huize Pang | 2022 | The striatum (The left dorsolateral putamen is an important area.) | Extrastriatal FC, Intrastriatal FC, mALFF, MD, volume, iron‐radiomic features | MRI (T1WI/DTI/rs-fMRI/SWI) | UPDRS III scores |
| 7 | Jingwen Li | 2022 | The whole brain | GLCM feature (Contrast),  GLSZM features (ZoneVariance, SmallAreaLowGrayLevelEmphasis),  GLCM feature (lmc1),  GLRLM feature (LongRunLowGrayLevelEmphasis),  Histogram features (Kurtosis, Uniformity) | MRI (DTI) | NA |
| 8 | Yun Soo Kim | 2022 | The putamen | GLCM features (Autocorrelation7, SumAverage4, JointAverage4, SumAverage7, JointAverage7, Imc24, MCC4, Autocorrelation4),  GLDM features (HighGrayLevelEmphasis, DependenceVariance),  GLRLM features (HighGrayLevelRunEmphasis, ShortRunHighGrayLevelEmphasis, GrayLevelNonUniformity) | MRI (T1WI/SWI) | NA |
| 9 | Jin Juan Kang | 2022 | SN, HCN, PUT | Shapes (elongation and least axis length),  gray level values (maximum, minimum, average and total energy),  the distribution of various gray level values (skewness, entropy, low gray level emphasis, etc.) | MRI (QSM) | NA |
| 10 | Xiao-Jun Guan | 2022 | SN，CN, PUT, GP, RN | Features reflecting iron distribution (the most important for diagnosing PD),  Texture features selected from FIRST,  Informative features of cortical volume | MRI (T1WI/QSM) | NA |
| 11 | Dafna Ben Bashat | 2022 | SN, RN | Mean, Skewness, Contrast, Correlation, Kurtosis, volume etc. | MRI (NM-MRI/T2WI) | NA |
| 12 | Xulian Zhang | 2021 | The whole brain DPDvs.HCs: DMN, ECN, VIN, AN, SMN, STM network, the left PrG and the left planum polare. NDPDvs.HCs: DMN, VIN, AN, SMN, AUN, VAN, ECN, salience network, BGN, the left juxtapositional lobule cortex, the left MTG, posterior division, the right temporal fusiform cortex, posterior division.  DPDvs.NDPD: DMN, VIN, STM, AN, BGN, SMN, salience network, ECN, VAN, AUN, the region of left subcallosal cortex. | RSFC, ALFF, ReHo, VMHC features | MRI (Rs-fMRI) | HAMD Score |
| 13 | Priyanka Tupe-Waghmare | 2021 | The brainstem, bilaterally the cerebellar gray matter, cerebellar white matter, caudate, putamen, pallidum, ventral diencephalon, thalamus, and nucleus accumbens, SNc. | PDvs.HC: First-order feature, GLCM and GLDM, thalamus.  PDvs.APS: The features for the ventral diencephalon and nucleus accumbens. | MRI (T1WI) | NA |
| 14 | Dong Sun | 2021 | Subcortical gray matter nuclei (the bilateral hippocampus, thalamus, PUT, GP, CN, nucleus accumbens and amygdala) | PDvs.HCs: The values of Contrast, SumVariance, SumVariance. (Radiomic features from the left hippocampus.) PIGDvs.TD: GLCM features (Cluster Prominence, Cluster Shade, Sum Variance, Autocorrelation, Contrast),  GLRLM feature (Short Run High Gray Level Emphasis) | MRI (T1WI) | NA |
| 15 | Dafa Shi | 2021 | The frontal, temporal, parietal, occipital, limbic lobes, the cerebellum and the thalamus. | The histogram features (Mean, Minimum, Maximum, Standard deviation, Median, Skewness, Kurtosis, 10th percentile) | MRI (T1WI/Rs-fMRI) | NA |
| 16 | Qingguo Ren | 2021 | SN | Histogram feature, form factor feature, RLM feature, GLCM feature | MRI (SWI) | NA |
| 17 | Xue-ning Li | 2021 | SN, RN | The radiomics signature extracted from bilateral SN and RN regions. | MRI (T2WI) | The plasma FAM19A5 level, gender, age |
| 18 | Xuehan Hu | 2021 | PUT, CN | First-order features (Skewness, RootMeanSquared, Maximum),  GLSZM features (SmallAreaEmphasis, SmallAreaLowGrayLevelEmphasis),  GLDM feature (SmallDependenceLowGrayLevelEmphasis),  GLCM feature (Idmn),  GLRLM features (RunLengthNonUniformity, LongRunEmphasis) | Hybrid 18F-FDG PET/MRI(T1WI, T2WI, T2/FLAIR, SWI, DWI) | DD, dysarthria, AF, age, sex, weight, pre-injection glucose levels, age at onset, hypermyotonia, asymmetric symptoms at onset, bradykinesia, limb tremor and ADC values, SUV values |
| 19 | Nikhil J. Dhinagar | 2021 | The whole brain | Textural features based on first-order statistical co-occurrence matrices | MRI (T1WI) | NA |
| 20 | Xuan Cao | 2021 | The whole brain (The radiomic features are mainly located in DMN, SMN, VIN.) | RSFC measures features | MRI (Rs-fMRI) | Gender, age, education level, MMSE and HAMD |
| 21 | Zhenyu Shu | 2020 | The whole brain (White Matter) | GLRLM features (GreyLevelNonuniformity_angle45_offset7, HighGreyLevelRunEmphasis_AllDirection_offset7_SD),  GLCM feature (GLCMEntropy_angle135_offset1),  Haralick feature (HaralickCorrelation_angle90_offset1) | MRI (T1WI) | Family history of PD, age, sex, nonmotor symptoms (IO, depression, RBD, EDS, CD) |
| 22 | Huize Pang | 2020 | Basal Nuclei (PUT, SN, CN, GP, RN, STN) | Radiomic features derived from the PUT had optimal value in differentiating IPD from MSA-P. As follows: Histogram feature (Std Deviance),  Textural feature (Correlation_angle0_offset1),  GLCM features (GLCMEntropv_AllDirection_offset7_SD, HaralickCorrelation_Alldirection_offset4, InverseDifferenceMoment_angle0_offset7, InverseDifferenceMoment_anglel35_offset7),  GLRLM feature (RunLengtliNonuniformity_AllDirection_offset 4_SD) | MRI (SWI) | UPDRS III scores |
| 23 | Panshi Liu | 2020 | The neostriatum (CN, PUT) | Image histogram, GLRLM features, GLCM features  (Vertl_GlevNonU_R appeared simultaneously in both the CN and PU radiomics signatures as an optimal feature.) | MRI (T2WI) | NA |
| 24 | Xuan Cao | 2020 | The whole brain The related brain regions note below: RSFCs (primarily located in the ECN, DMN, AN, VIN and SMN.) mALFF (primarily located in the left superior temporal gyrus, posterior division.)  mReHo (primarily located in the left parahippocampal gyrus, posterior division, right thalamus and left pallidum.)  VMHC (primarily located in the right temporal fusiform cortex, anterior division.) GM volume (primarily located in the right inferior temporal gyrus, anterior division and the right accumbens.) | mALFF, mReHo, RSFC, VMHC, GM volume feature | MRI (Rs-fMRI) | HAMD Score |
| 25 | Bin Xiao | 2019 | SN | GLRLM (Median, RunEntropy, LongRunEmphasis),  GLCM (ClusterShade, IDN, DifferenceVariance, DifferenceEntropy, Correlation),  First-order (Mean, Median, Skewness),  GLDM (DependenceVariance, DependenceEntropy)  CNN-derived features | MRI (QSM) | NA |
| 26 | Yue Wu | 2019 | SFG, MFG, SMA, OG, CN, PUT, GP, thalamus, ITG, cerebellum, pons. | LGZE, Skewness, LRHGE, Variance, Entropy, LZHGE, Strength, Coasenes, Kurtosis, LZLGE feature | PET (18F-FDG PET) | NA |
| 27 | Sumeet Shinde | 2019 | SNc | GLRLM, non-uniformity measures, surface-volume ratios, GLDM features Features extracted automatically based on CNN. | MRI (NMS-MRI) | NA |
| 28 | Zenghui Cheng | 2019 | SN | First-order feature (10 Percentile), Median, GLRLM feature (LRunLGREmphs), GLSZM feature (GryLvNonUniS), Shape-Volume | MRI (QSM) | NA |

Abbreviations: SN, substantia nigra; SNc, substantia nigra pars compacta; PUT, putamen; GP, globus pallidus; CN, caudate nucleus; HCN, head of caudate nucleus; RN, red nucleus; STN, subthalamic nucleus; DMN, default mode network; ECN, executive control network; VIN, visual network; AN, affective network; SMN, sensorimotor network; STM, short‐term memory; AUN, automatic urban network; VAN, ventral attention network; BGN, basal ganglia network; SFG, superior frontal gyrus; MFG, middle frontal gyrus; IFG, inferior frontal gyrus; STG, superior temporal gyrus; MTG, middle temporal gyrus; ITG, inferior temporal gyrus; PrG, precentral gyrus; PoG, postcentral gyrus; PCL, paracentral lobule; PCun, precuneus; SPL, superior parietal lobule; SMA, supplementary motor area; PhG, parahippocampal gyrus; OG, occipital gyrus; mALFF, mean amplitude of low‐frequency fluctuation; ReHo, regional homogeneity; RSFC, resting-state functional connectivity; VMHC, voxel-mirrored homotopic connectivity; GM, gray matter; MD, mean diffusivity; NMS-MRI, Neuromelanin sensitive magnetic resonance imaging; T1WI, T1-weighted imaging; T2WI, T2-weighted imaging; DTI, diffusion tensor imaging; rs-fMRI, resting-state functional magnetic resonance imaging; SWI, susceptibility-weighted imaging; GLCM, Gray Level Co-occurrence Matrix; GLDM, Gray Level Difference Matrix; GLRLM, Gray-Level Run-Length Matrix; GLSZM, Gray Level Size Zone Matrix; NGLDM, Neighboring Gray Level Dependence Matrix; NGTDM, Neighbouring Gray Tone Difference Matrix; LGZE, low gray-level zone emphasis; LRHGE, long-run high gray-level emphasis; LZHGE, large zone high-gray-level emphasis; LZLGE, large zone low-gray-level emphasis; UPDRSIII, Motor scores of the unified Parkinson's Disease Rating Scale; HAMD, Hamilton depression rating scale; MMSE, mini-mental state examination; DD, Disease duration; AF, autonomic failure; IO, impaired olfaction; RBD, Rapid eye movement sleep behavior disorder; EDS, excessive daytime sleepiness; CD, cognitive decline; L, left; R, right;

**Supplementary Table S5.** Meta-analysis results of c-index for PD diagnosis based on radiomics-based machine learning.

| Model | Training set | | | Validation set | | |
| --- | --- | --- | --- | --- | --- | --- |
|  | Number | c-index(95%CI) | I²(%) | Number | c-index(95%CI) | I²(%) |
| SVM | 16 | 0.872[0.834~0.910] | 90.7 | 28 | 0.864[0.829~0.899] | 92.2 |
| LR | 11 | 0.813[0.729~0.898] | 96.8 | 12 | 0.796[0.723~0.869] | 88.7 |
| RF | 5 | 0.842[0.731~0.953] | 96.6 | 9 | 0.669[0.545~0.793] | 83.2 |
| LASSO | 3 | 0.940[0.892~0.987] | 71.8 | 5 | 0.902[0.818~0.986] | 82.7 |
| ANN | 2 | 0.813[0.729~0.898] | 0.0 | 2 | 0.796[0.723~0.869] | 0.0 |
| CNN | 2 | 0.925[0.893~0.957] | 0.0 | 3 | 0.779[0.615~0.943] | 80.8 |
| KNN | 1 | 0.790[0.715~0.865] | NA | 7 | 0.896[0.826~0.965] | 87.8 |
| DT | 1 | 0.897[0.861~0.933] | NA | 6 | 0.962[0.944~0.979] | 0.0 |
| Bayes | 1 | 0.903[0.868~0.938] | NA | NA | NA | NA |
| LDA | NA | NA | NA | 6 | 0.986[0.977~0.996] | 23.6 |
| overall | 42 | 0.862[0.833~0.891] | 94.6 | 78 | 0.871[0.853~0.890] | 92.4 |

Note: NA, Not Available.

**Supplementary Table S6.** Meta-analysis results of c-index for differential diagnosis between PD and APS based on radiomics-based machine learning.

| Model | Training set | | | Validation set | | |
| --- | --- | --- | --- | --- | --- | --- |
|  | Number | c-index(95%CI) | I²(%) | Number | c-index(95%CI) | I²(%) |
| SVM | 10 | 0.895[0.856~0.935] | 90.7 | 14 | 0.905[0.875~0.935] | 54.8 |
| LR | 2 | 0.952[0.857~1.000] | 77.8 | 2 | 0.942[0.812~1.000] | 63.4 |
| RF | 3 | 0.848[0.748~0.949] | 80.9 | 4 | 0.894[0.794~0.994] | 79.7 |
| LASSO | 1 | 0.971[0.919~1.000] | NA | 1 | 0.957[0.859~1.000] | NA |
| ANN | 3 | 0.863[0.810~0.917] | 31.9 | 3 | 0.857[0.775~0.938] | 27.8 |
| CNN | 4 | 0.914[0.865~0.963] | 77.7 | 1 | 0.911[0.809~1.000] | NA |
| KNN | 3 | 0.828[0.721~0.936] | 81.3 | 3 | 0.832[0.722~0.941] | 56.0 |
| DT | 3 | 0.754[0.629~0.878] | 79.9 | 3 | 0.766[0.635~0.898] | 58.8 |
| ADA | 3 | 0.837[0.731~0.944] | 81.9 | 3 | 0.845[0.741~0.950] | 55.1 |
| QDA | 3 | 0.846[0.728~0.964] | 86.7 | 3 | 0.872[0.784~0.961] | 46.3 |
| GNB | 3 | 0.863[0.757~0.969] | 85.5 | 3 | 0.872[0.767~0.976] | 63.5 |
| GP | 3 | 0.757[0.561~0.953] | 92.8 | 3 | 0.729[0.498~0.960] | 87.1 |
| overall | 41 | 0.866[0.843~0.889] | 86.4 | 43 | 0.879[0.854~0.903] | 67.6 |

**Supplementary Table S7.** Meta-analysis results of c-index for differential diagnosis between PD and MSA based on radiomics-based machine learning.

| Model | Training set | | | Validation set | | |
| --- | --- | --- | --- | --- | --- | --- |
|  | Number | c-index(95%CI) | I²(%) | Number | c-index(95%CI) | I²(%) |
| SVM | 8 | 0.882[0.836~0.928] | 92.3 | 12 | 0.901[0.867~0.934] | 58.7 |
| LR | 2 | 0.952[0.857~1.000] | 77.8 | 2 | 0.942[0.812~1.000] | 63.4 |
| RF | 2 | 0.820[0.626~1.000] | 90.2 | 2 | 0.820[0.628~1.000] | 76.4 |
| LASSO | 1 | 0.971[0.893~0.997] | NA | 1 | 0.957[0.859~1.000] | NA |
| ANN | 2 | 0.861[0.769~0.953] | 64.8 | 2 | 0.866[0.745~0.986] | 52.5 |
| KNN | 2 | 0.790[0.597~0.983] | 88.8 | 2 | 0.795[0.594~0.995] | 75.9 |
| DT | 2 | 0.754[0.544~0.964] | 89.4 | 2 | 0.768[0.551~0.985] | 77.5 |
| ADA | 2 | 0.809[0.605~1.000] | 90.7 | 2 | 0.812[0.616~1.000] | 76.6 |
| QDA | 2 | 0.817[0.583~1.000] | 93.4 | 2 | 0.845[0.676~1.000] | 72.6 |
| GNB | 2 | 0.823[0.609~1.000] | 92.2 | 2 | 0.830[0.623~1.000] | 80.5 |
| GP | 2 | 0.818[0.600~1.000] | 92.3 | 2 | 0.828[0.638~1.000] | 76.6 |
| overall | 27 | 0.857[0.827~0.887] | 88.8 | 31 | 0.878[0.852~0.905] | 60.7 |

**Supplementary Table S8.** Meta-analysis results of c-index for differential diagnosis between PD and PSP based on radiomics-based machine learning.

| Model | Training set | | | Validation set | | |
| --- | --- | --- | --- | --- | --- | --- |
|  | Number | c-index(95%CI) | I²(%) | Number | c-index(95%CI) | I²(%) |
| SVM | 2 | 0.942[0.906~0.978] | 0.0 | 2 | 0.934[0.875~0.993] | 0.0 |
| RF | 1 | 0.890[0.819~0.960] | NA | 1 | 0.887[0.778~0.996] | NA |
| ANN | 1 | 0.856[0.777~0.936] | NA | 1 | 0.814[0.677~0.952] | NA |
| KNN | 1 | 0.890[0.819~0.960] | NA | 1 | 0.876[0.762~0.990] | NA |
| DT | 1 | 0.745[0.643~0.848] | NA | 1 | 0.743[0.586~0.901] | NA |
| ADA | 1 | 0.879[0.805~0.952] | NA | 1 | 0.881[0.768~0.993] | NA |
| QDA | 1 | 0.887[0.816~0.958] | NA | 1 | 0.895[0.790~1.000] | NA |
| GNB | 1 | 0.920[0.860~0.980] | NA | 1 | 0.915[0.821~1.000] | NA |
| GP | 1 | 0.631[0.515~0.746] | NA | 1 | 0.522[0.337~0.707] | NA |
| overall | 10 | 0.871[0.826~0.915] | 75.4 | 10 | 0.863[0.808~0.918] | 59.1 |

**Supplementary Table S9.** Meta-analysis results of c-index for differential diagnosis between TD and PIGD based on radiomics-based machine learning.

| Model | Training set | | | Validation set | | |
| --- | --- | --- | --- | --- | --- | --- |
|  | Number | c-index(95%CI) | I²(%) | Number | c-index(95%CI) | I²(%) |
| SVM | 1 | 0.889[0.824~0.954] | NA | 1 | 0.833[0.668~0.998] | NA |
| LR | 1 | 0.890[0.825~0.955] | NA | 1 | 0.819[0.648~0.990] | NA |
| ANN | 1 | 0.897[0.834~0.960] | NA | 1 | 0.812[0.638~0.986] | NA |
| overall | 3 | 0.892[0.855~0.929] | 0.0 | 3 | 0.822[0.724~0.920] | 0.0 |

**Supplementary Table S10.** Meta-analysis results of sensitivity and specificity for differential diagnosis between TD and PIGD based on radiomics-based machine learning.

| Model | Training set | | | | | Validation set | | | | |
| --- | --- | --- | --- | --- | --- | --- | --- | --- | --- | --- |
|  | Number | Sen(95%CI) | I²(%) | Spe(95%CI) | I²(%) | Number | Sen(95%CI) | I²(%) | Spe(95%CI) | I²(%) |
| SVM | 1 | 0.88 | NA | 0.80 | NA | 1 | 0.75 | NA | 0.83 | NA |
| LR | 1 | 0.88 | NA | 0.82 | NA | 1 | 0.88 | NA | 0.66 | NA |
| ANN | 1 | 0.85 | NA | 0.77 | NA | 1 | 0.88 | NA | 0.72 | NA |
| overall | 3 | 0.85~0.88 | NA | 0.77~0.82 | NA | 3 | 0.75~0.88 | NA | 0.66~0.83 | NA |

**Differential diagnosis PD with and without depression**

In terms of the differential diagnosis of PD with and without depression, only one model (LR) was included in the training set due to the limited number of the included studies. The c-index in the training set was 0.937 (95% CI: 0.874-0.999). There were three models in the validation set, with a pooled c-index of 0.948 (95% CI: 0.845-1.000). The sensitivity and specificity in the training set were 0.82 and 0.90, respectively. The pooled sensitivity and specificity in the validation set were 1.00 and 0.77-1.00, respectively. These results are displayed in Tables S11 and S12.

**Supplementary Table S11.** Meta-analysis results of c-index for differential diagnosis between DPD and NDPD based on radiomics-based machine learning.

| Model | Training set | | | Validation set | | |
| --- | --- | --- | --- | --- | --- | --- |
|  | Number | c-index(95%CI) | I²(%) | Number | c-index(95%CI) | I²(%) |
| SVM | NA | NA | NA | 1 | 0.860[0.685~1.000] | NA |
| LASSO | NA | NA | NA | 1 | 0.980[0.913~1.000] | NA |
| RF | NA | NA | NA | 1 | 0.980[0.913~1.000] | NA |
| LR | 1 | 0.937[0.874~0.999] | NA | NA | NA | NA |
| overall | 1 | 0.937[0.874~0.999] | NA | 3 | 0.948[0.845~1.000] | 36.8 |

**Supplementary Table S12.** Meta-analysis results of sensitivity and specificity for differential diagnosis between DPD and NDPD based on radiomics-based machine learning.

| Model | Training set | | | | | Validation set | | | | |
| --- | --- | --- | --- | --- | --- | --- | --- | --- | --- | --- |
|  | Number | Sen(95%CI) | I²(%) | Spe(95%CI) | I²(%) | Number | Sen(95%CI) | I²(%) | Spe(95%CI) | I²(%) |
| SVM | NA | NA | NA | NA | NA | 1 | 1.00 | NA | 0.77 | NA |
| LASSO | NA | NA | NA | NA | NA | 1 | 1.00 | NA | 0.92 | NA |
| RF | NA | NA | NA | NA | NA | 1 | 1.00 | NA | 1.00 | NA |
| LR | 1 | 0.82 | NA | 0.90 | NA | NA | NA | NA | NA | NA |
| overall | 1 | 0.82 | NA | 0.90 | NA | 3 | 1.00 | NA | 0.77~1.00 | NA |


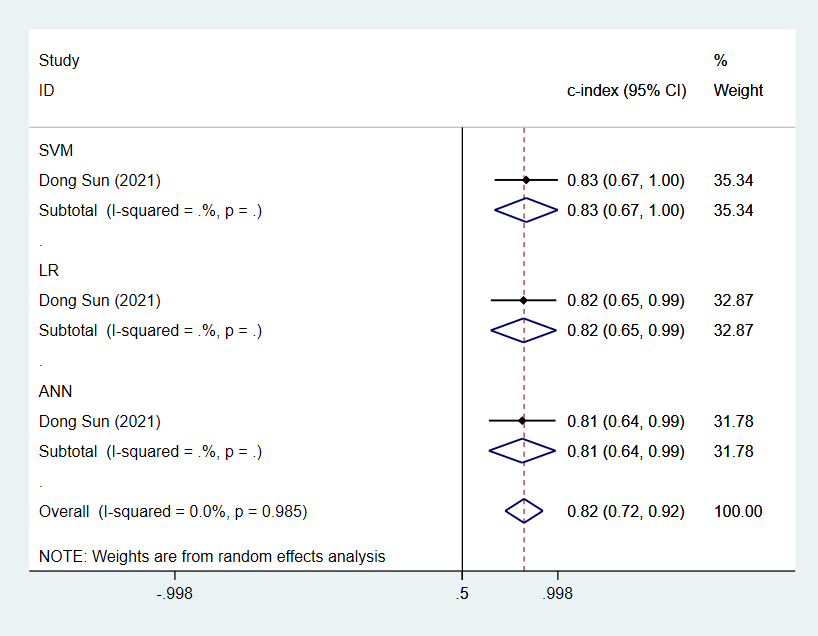


**Supplementary Figure S1.** Meta-analysis results of c-index for differential diagnosis between TD and PIGD based on radiomics-based machine learning (Validation set).


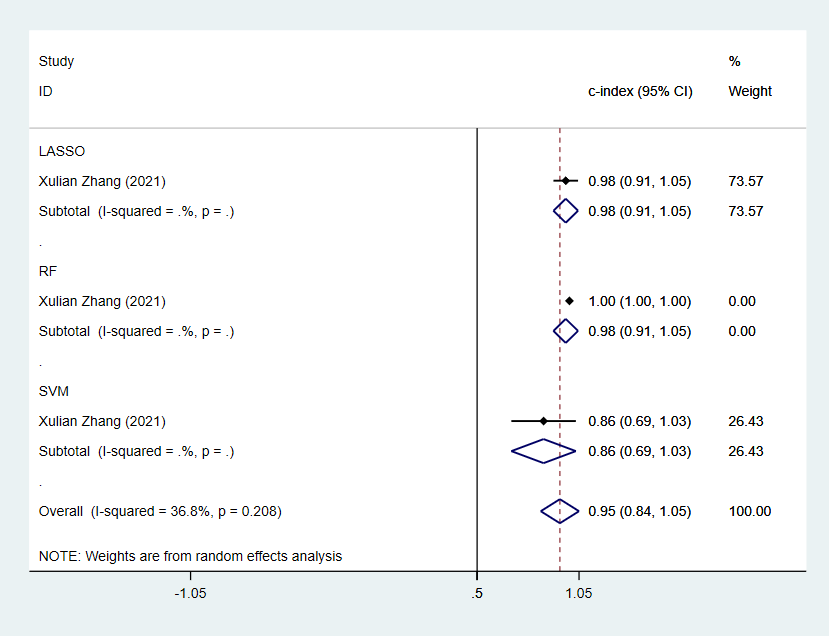


**Supplementary Figure S2.** Meta-analysis results of c-index for differential diagnosis between DPD and NDPD based on radiomics-based machine learning (Validation set).


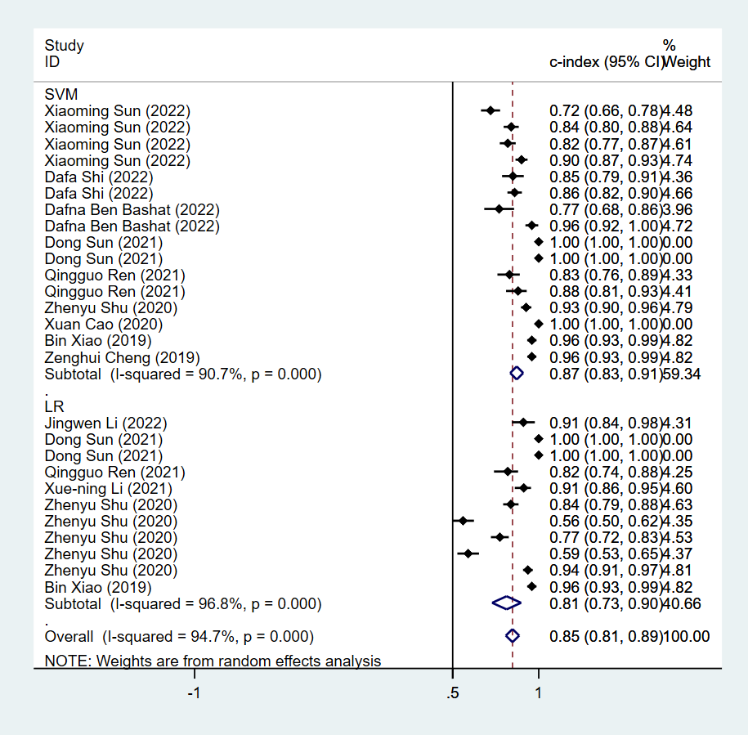

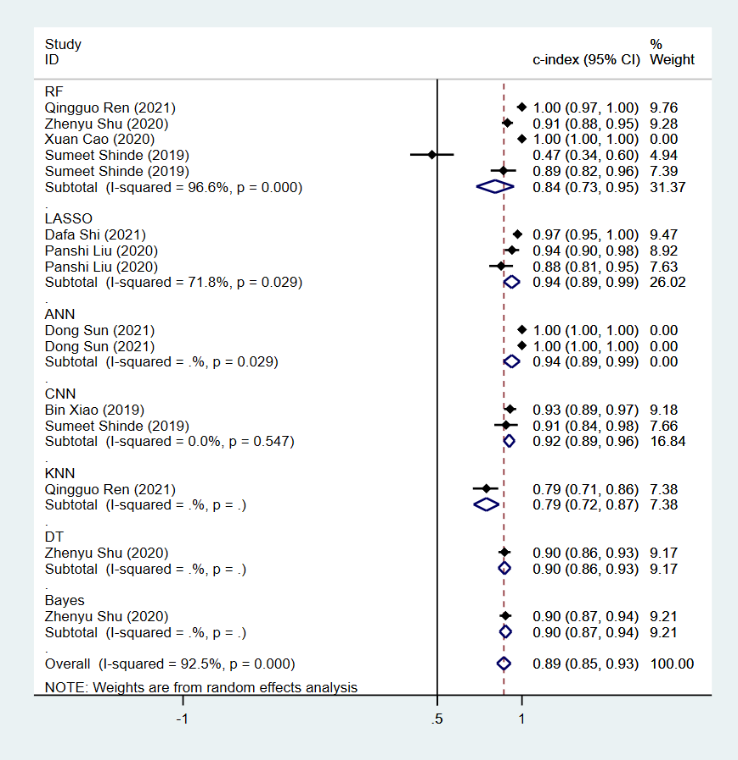


**Supplementary Figure S3.** Meta-analysis results of c-index for PD diagnosis based on radiomics-based machine learning (Training set).


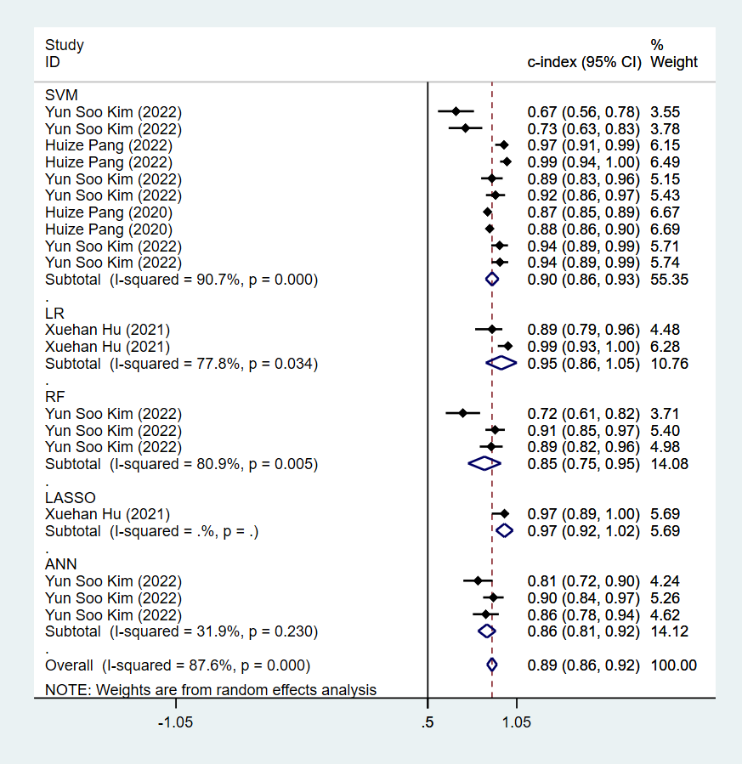

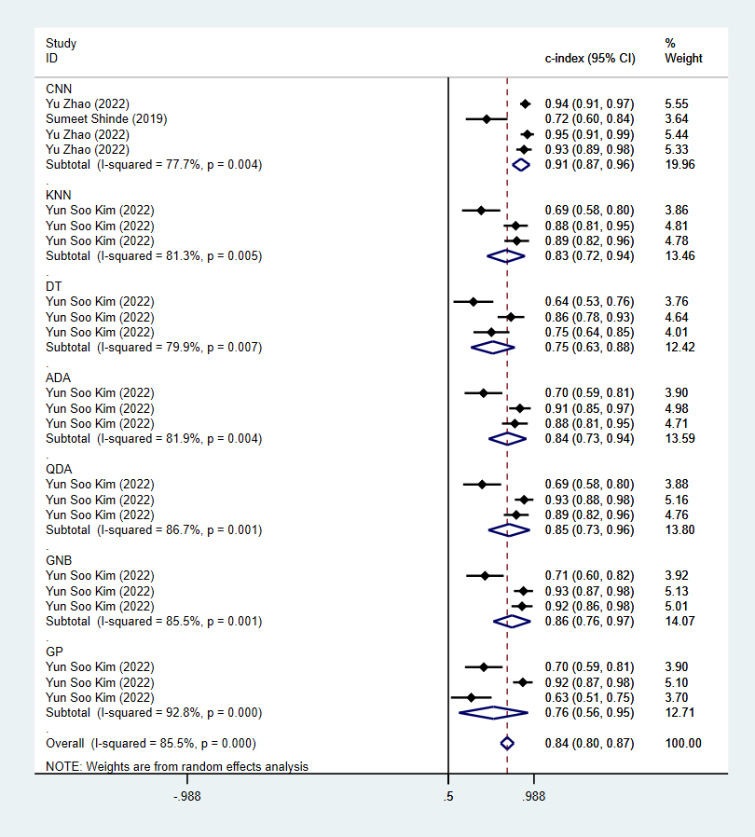


**Supplementary Figure S4.** Meta-analysis results of c-index for differential diagnosis between PD and APS based on radiomics-based machine learning (Training set).


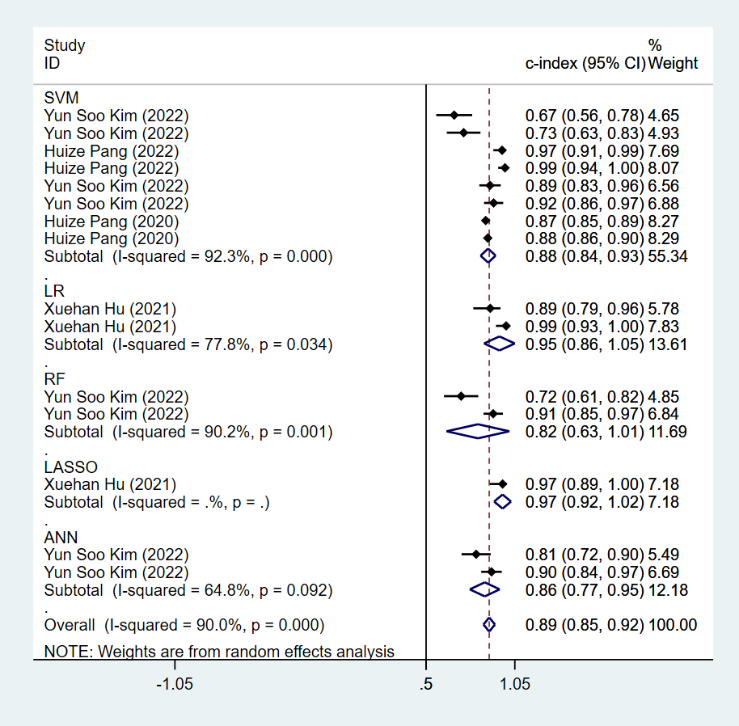

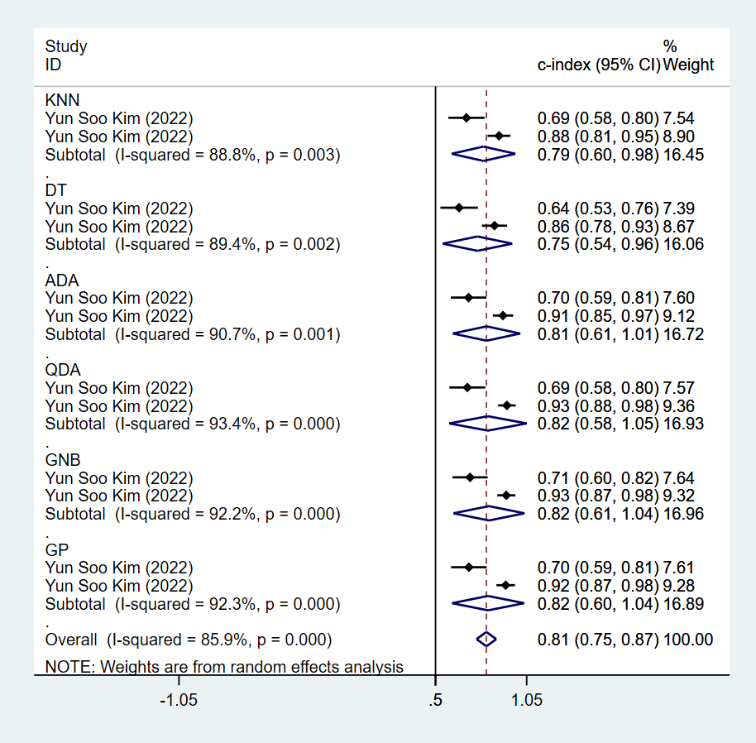


**Supplementary Figure S5.** Meta-analysis results of c-index for differential diagnosis between PD and MSA based on radiomics-based machine learning (Training set).


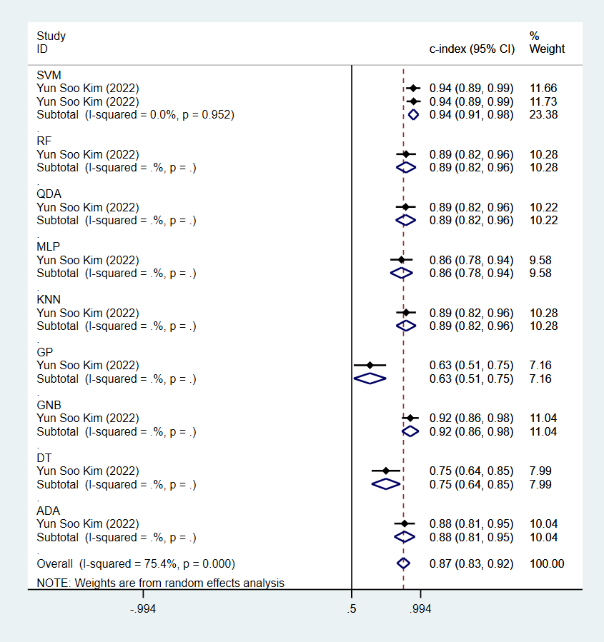


**Supplementary Figure S6.** Meta-analysis results of c-index for differential diagnosis between PD and PSP based on radiomics-based machine learning (Training set).


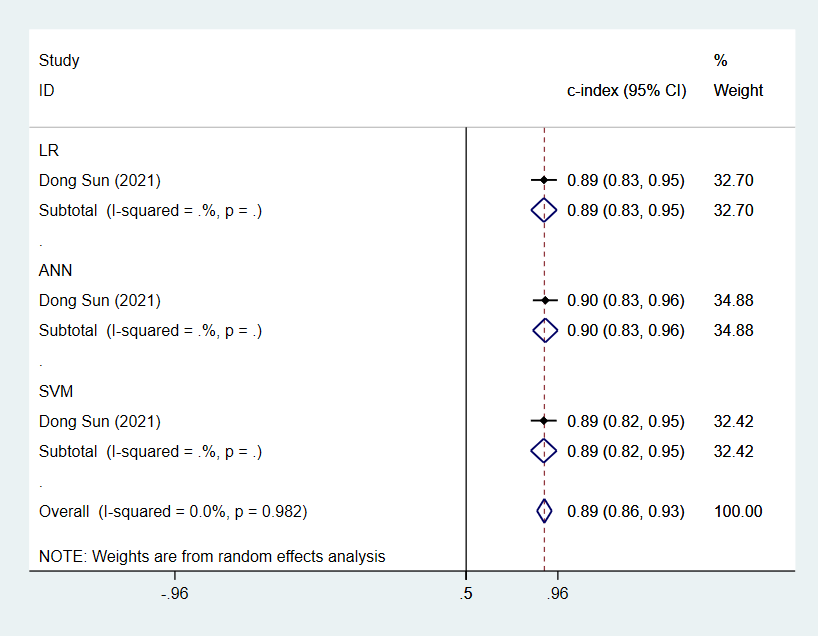


**Supplementary Figure S7.** Meta-analysis results of c-index for differential diagnosis between TD and PIGD based on radiomics-based machine learning (Training set).


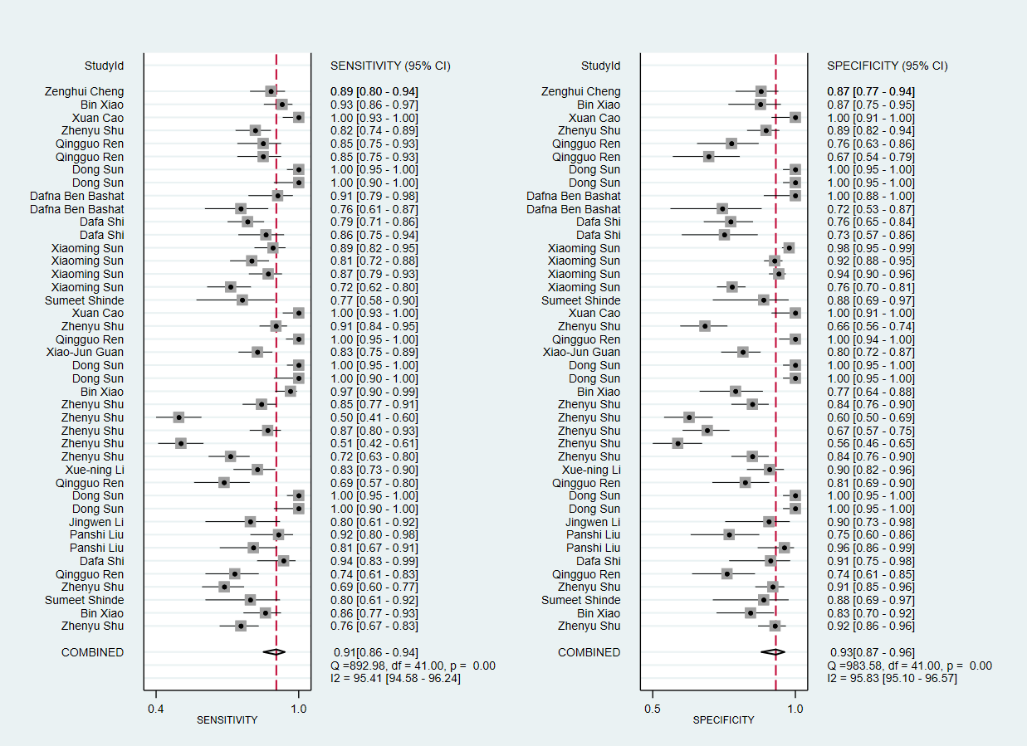


(a). Training set


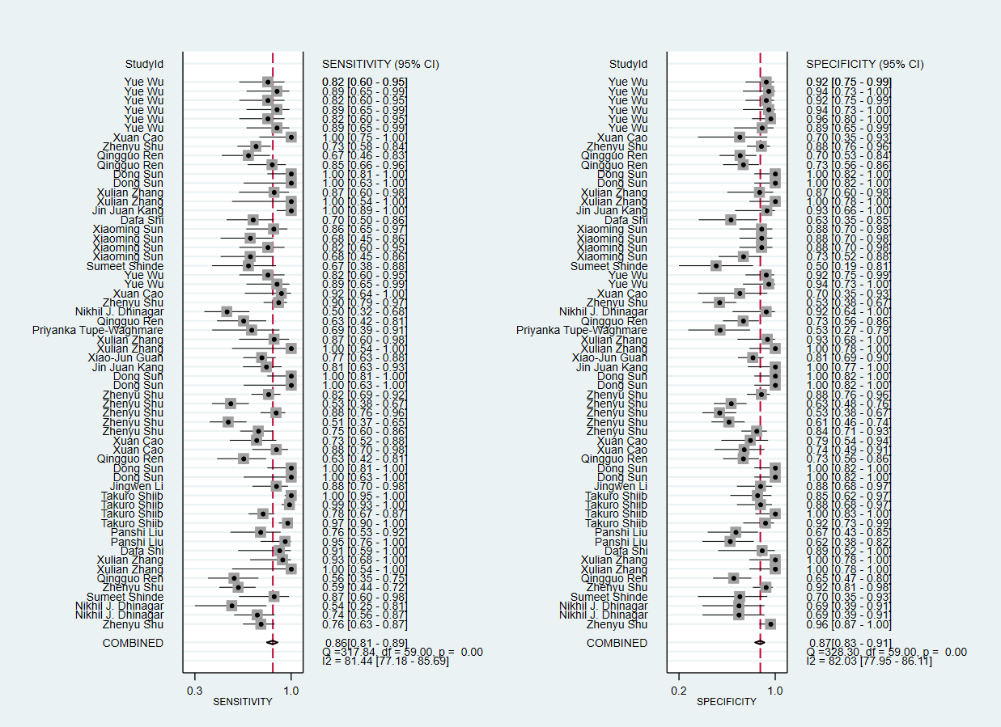


(b). Validation set

**Supplementary Figure S8.** Meta-analysis results of sensitivity and specificity for PD diagnosis based on radiomics-based machine learning.


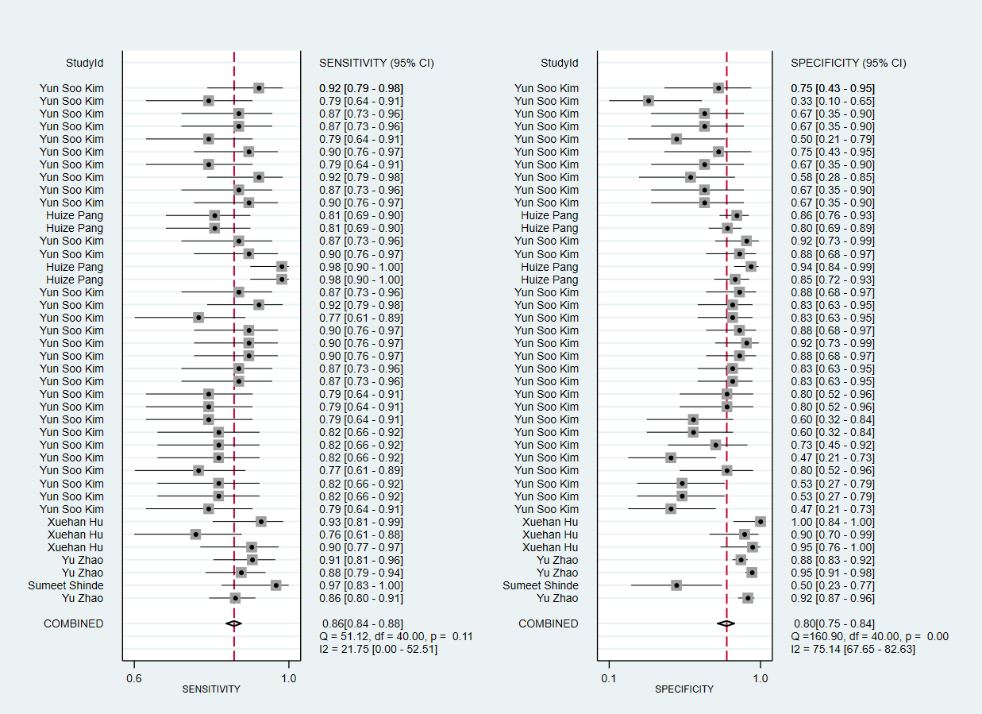


(a). Training set


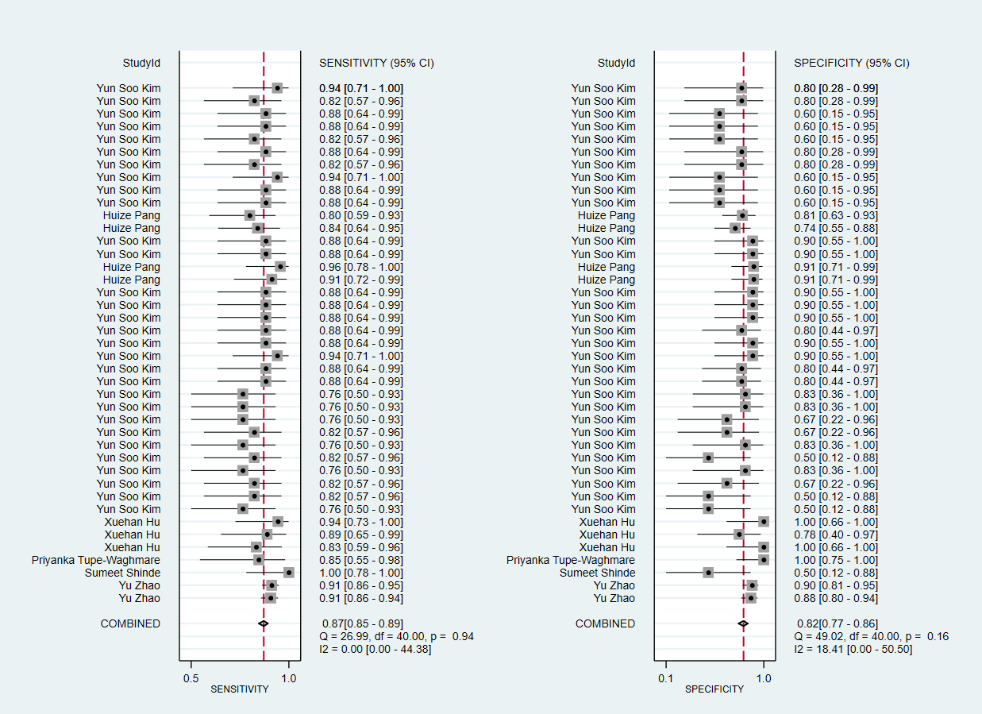


(b). Validation set

**Supplementary Figure S9.** Meta-analysis results of sensitivity and specificity for differential diagnosis between PD and APS based on radiomics-based machine learning.


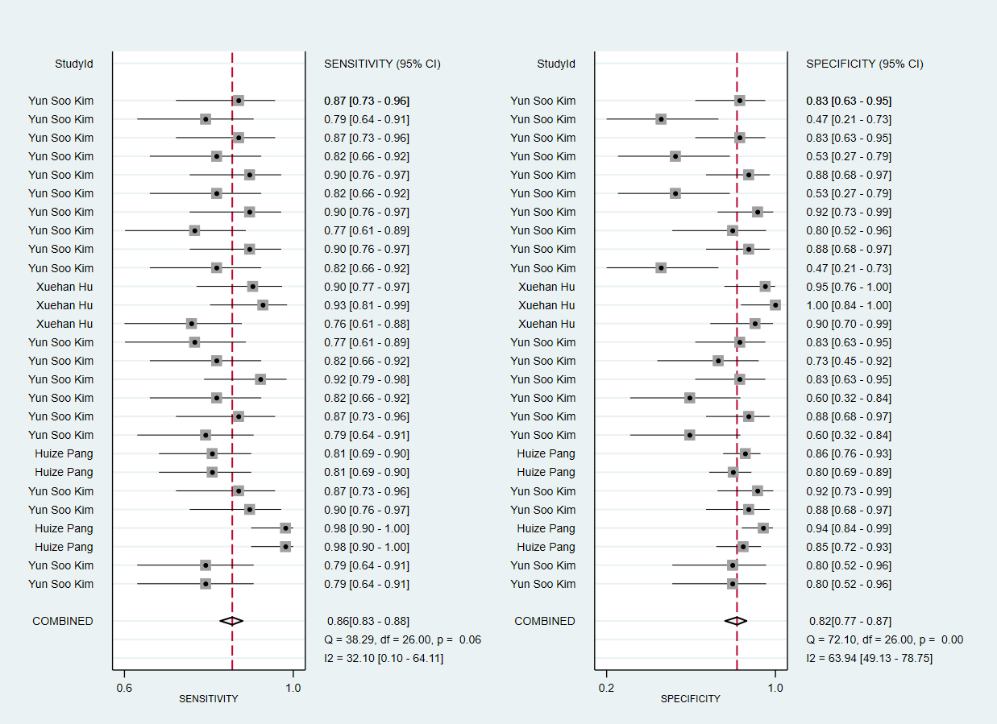


(a). Training set


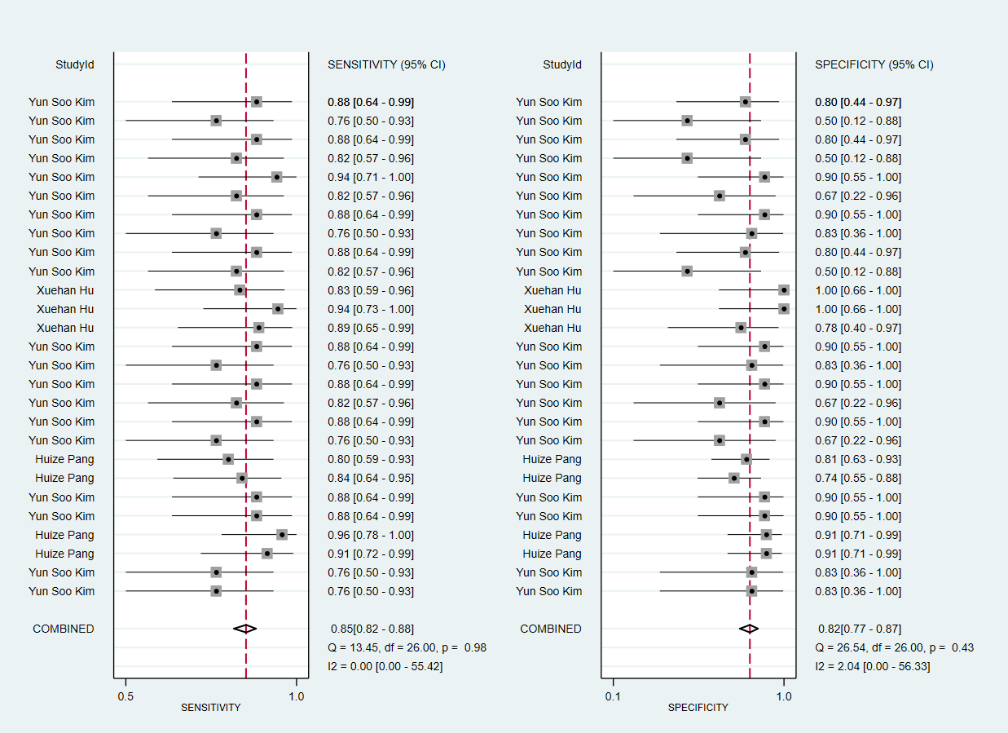


(b). Validation set

**Supplementary Figure S10.** Meta-analysis results of sensitivity and specificity for differential diagnosis between PD and MSA based on radiomics-based machine learning.


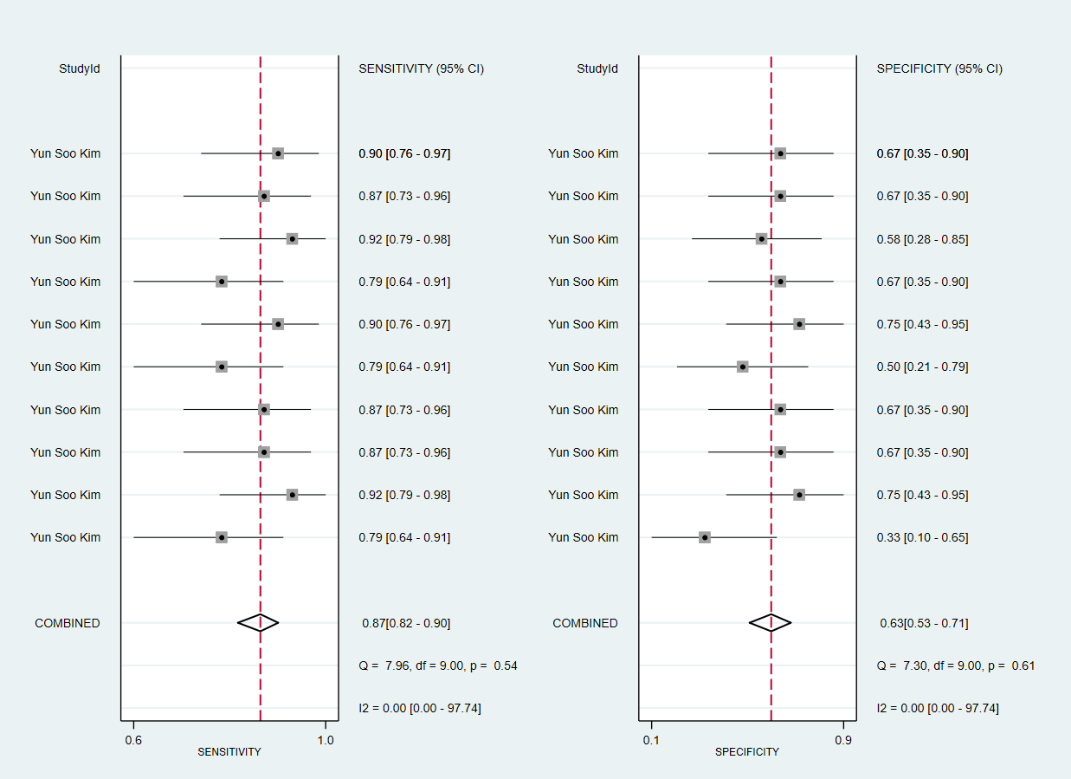


(a). Training set


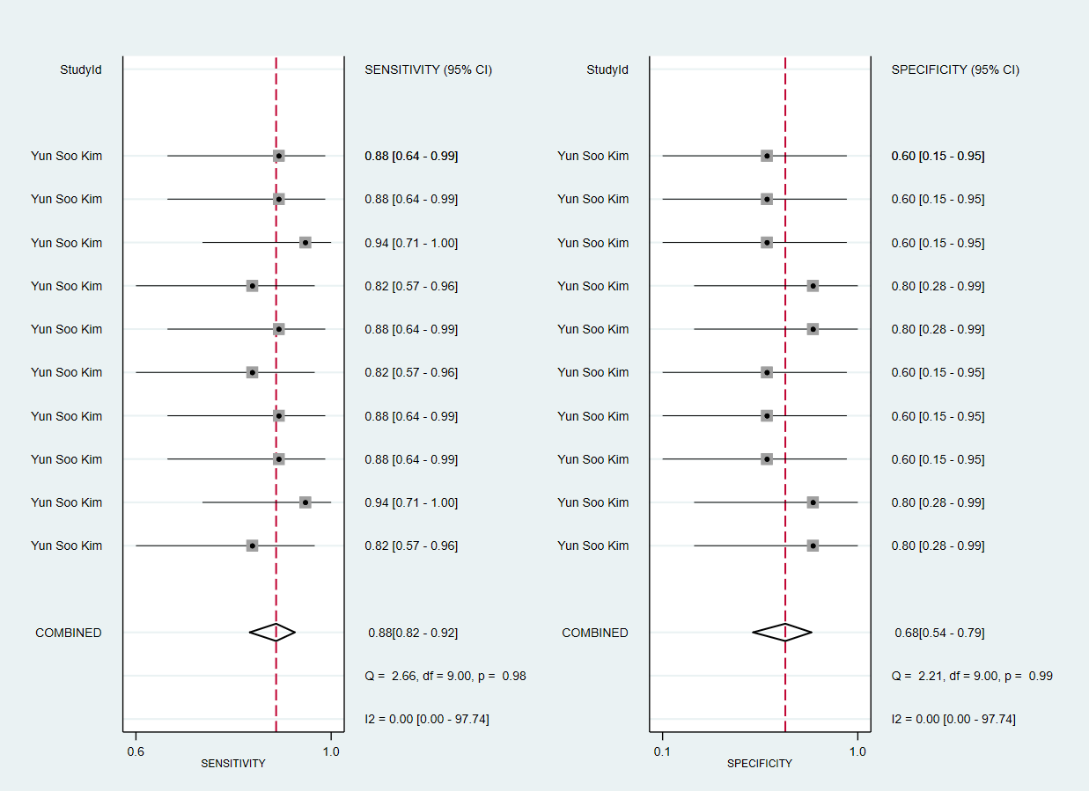


(b). Validation set

**Supplementary Figure S11.** Meta-analysis results of sensitivity and specificity for differential diagnosis between PD and PSP based on radiomics-based machine learning.
